# Sequential biases in accumulating evidence

**DOI:** 10.1002/jrsm.1185

**Published:** 2015-12-01

**Authors:** Elena Kulinskaya, Richard Huggins, Samson Henry Dogo

**Affiliations:** ^1^ School of Computing SciencesUniversity of East AngliaNorwichNR4 7TJUK; ^2^ Department of Mathematics and StatisticsUniversity of MelbourneMelbourneAustralia

**Keywords:** accumulating evidence, cumulative meta‐analysis, sequential meta‐analysis, sequential bias

## Abstract

Whilst it is common in clinical trials to use the results of tests at one phase to decide whether to continue to the next phase and to subsequently design the next phase, we show that this can lead to biased results in evidence synthesis. Two new kinds of bias associated with accumulating evidence, termed ‘sequential decision bias’ and ‘sequential design bias’, are identified. Both kinds of bias are the result of making decisions on the usefulness of a new study, or its design, based on the previous studies. Sequential decision bias is determined by the correlation between the value of the current estimated effect and the probability of conducting an additional study. Sequential design bias arises from using the estimated value instead of the clinically relevant value of an effect in sample size calculations. We considered both the fixed‐effect and the random‐effects models of meta‐analysis and demonstrated analytically and by simulations that in both settings the problems due to sequential biases are apparent. According to our simulations, the sequential biases increase with increased heterogeneity. Minimisation of sequential biases arises as a new and important research area necessary for successful evidence‐based approaches to the development of science. © 2015 The Authors. *Research Synthesis Methods* Published by John Wiley & Sons Ltd.

For with much wisdom comes much sorrow; the more knowledge, the more grief.

Ecclesiastes 1:18

## 1 Introduction

The idea that the results of previous meta‐analyses should be used for design of new trials is widely recognised. For example, the UK Medical Research Council requires a comprehensive review of existing evidence before funding trials (Glasziou *et al*., 2006). The guidelines of several medical journals including the Journal of American Medical Association and the Lancet state that all reports of clinical trials must include a summary of previous research findings and explain how the new trial affects this summary with direct reference to existing meta‐analysis (Goudie *et al*., 2010).

There are two ways of using prior analyses to inform further research. The first is using prior information in making the decision to conduct a new trial (sequential decision). The second is using prior analyses and systematic reviews to design the next trial (sequential design). That is, both the decision to conduct an experiment and the subsequent design of this experiment may depend on the results of previous experiments, and after the new experiment is conducted, the results are combined in an updated meta‐analysis.

Sequential and cumulative meta‐analyses are established techniques in both fixed‐effects and random‐effects meta‐analyses. See Whitehead (1997), Higgins *et al*. (2011), van der Tweel and Bollen (2010) to name a very few. Often, in a sequential analysis, when the results seem promising, after each trial, the only decision is whether or not to add the next trial in a sequence of independent trials. Whilst not advocating the approach and remarking on its inherent flaws, van der Tweel and Bollen (2010) noted that

“The usual approach is to repeatedly test the null hypothesis of equal effectiveness of two treatments on the cumulative data. If the test result is not statistically significant, a new trial is added and the test is repeated”.

But the sequence of trials may also be stopped for futility, and a systematic review would then reach the conclusion that a new trial is unnecessary (Goudie *et al*., 2010).

The setting explored here differs from the standard sequential meta‐analysis setting in that after *K* studies are accumulated and their results meta‐analysed, a meta‐analyst has an active role in the decision‐making and the design of the subsequent, (*K* + 1)‐st study, aiming at the definitive meta‐analysis of the (*K* + 1) studies. No direct involvement beyond this planned study is assumed. We examine the effect of prior knowledge on decision‐making in Section 2, where we show that if the probability of proceeding to a new trial is correlated with the current estimate of the effect size, the new combined estimator from a meta‐analysis will be biased (sequential decision bias).

Systematic reviews are often used to inform study design (Cooper *et al*., 2005). The sample size of the new study is calculated to yield a given type‐I error rate, typically 5%, and power, typically 80%, to detect a given effect size. ‘Researchers use the review to …estimate sample sizes’ (Mulrow, 1994). Or,

“In this context, we would wish to have answers to questions such as how a new study will influence the result of the meta‐analysis or how much more information might be needed to make the meta‐analysis conclusive (Roloff *et al*., 2013)”.

‘A new trial may, thus, be designed so as to achieve a decisive result when it is added to an existing meta‐analysis.’ (Goudie *et al*., 2010). We demonstrate in Section 3 that if the previous experiment is used to determine the effect size used in the sample size calculations in this fashion, then the combined estimator is biased. To illustrate these effects, we consider both fixed‐effect and random‐effects meta‐analyses analytically and by simulation, and in both settings, the problems become apparent. In Section 4, we illustrate the arising biases using as an example the systematic review by Johnson (1993). Similar biases appear and are well understood in group‐sequential and adaptive clinical trials. In this area, the existence of sequential bias is widely recognised and the means of adjustment for this bias have been developed (Denne, 2000; Emerson & Fleming, 1990; Kirby *et al*., 2012; Li *et al*., 2002; Whitehead, 1986). Discussion is given in Section 5 where, among other issues, we discuss similarities and differences between our setting and that of drug development. Proofs of some technical results, description of our simulations and additional figures depicting the simulation results are given in the Supporting Information.

## 2 Sequential decision bias

### 2.1 Bias

To illustrate sequential decision bias, we consider the following simple situation. Suppose there is a study that had estimated the effect of interest, *θ*, by ${\hat{\theta }}_{1}$ and its variance by $s_{1}^{2}$. A researcher is considering the usefulness of running another study. Suppose that the probability of running this new study $p_{\left(1\right)}=p\left({\hat{\theta }}_{1},s_{1}^{2},\theta_{0}\right)$ is a function of the estimated effect, its precision and the effect of clinical interest *θ*_0_ that is the same in both studies. In general, we denote by ${\hat{\theta }}_{i}$ and $s_{i}^{2}$ the effect and the estimated variance from the *i*th study, *i* ≥ 1. We also adopt sequential notation using bracketed subscripts, so that ${\hat{\theta }}_{\left(i\right)}$ is the meta‐analytically combined effect from the first *i* studies and $s_{\left(i\right)}^{2}$ is its estimated variance. For the first study, ${\hat{\theta }}_{1}={\hat{\theta }}_{\left(1\right)}$. We denote by *ω*_*i*_ the normalised inverse variance weights for ${\hat{\theta }}_{\left(i\right)}$, i.e. *ω*_1_ + *ω*_2_ + ⋯ *ω*_*i*_ = 1. If the second study is conducted, then the combined effect ${\hat{\theta }}_{\left(2\right)}$ is

$${\hat{\theta }}_{\left(2\right)}=\left\{\begin{array}{cc} \omega_{1}{\hat{\theta }}_{1}+\omega_{2}{\hat{\theta }}_{2}, & \;\text{with probability}\;p\left({\hat{\theta }}_{1},s_{1}^{2},\theta_{0}\right),\\ {\hat{\theta }}_{1}, & \;\text{with probability}\;1-p\left({\hat{\theta }}_{1},s_{1}^{2},\theta_{0}\right)\text{.} \end{array}\right.$$

The main results on sequential design bias are given below in Equations (1), (2), (3), and their proofs are provided in Section A of the Supporting Information.

To derive the results on bias analytically, we assume that ${\hat{\theta }}_{1}$ and ${\hat{\theta }}_{2}$ are independent and also that the weights are either non‐random or at least independent of the estimated effects. This strong assumption, although common in meta‐analysis, is fully satisfied for the weights based on inverse sample variances only when the effects are the sample means of continuous outcomes. It is also approximately true in the fixed‐effect model when the studies in the meta‐analysis are sufficiently large. To demonstrate that sequential decision bias arises in a quite general setting, in Section 2.2, we also provide simulation results for several decision‐making models in random‐effects meta‐analysis. All our simulations are based on 10 000 values of ${\hat{\theta }}_{1}$, providing precision to the second decimal place for estimated biases.

Under the aforementioned assumptions, we prove that the expected value of the combined estimator is

(1) $$E\left({\hat{\theta }}_{\left(2\right)}\right)=\theta +\left(\omega_{1}-1\right)Cov\left(p\left({\hat{\theta }}_{1},s_{1}^{2},\theta_{0}\right),{\hat{\theta }}_{1}\right)\text{.}$$

From Equation 1, we see, for example, that if the value of current estimate ${\hat{\theta }}_{1}$ is positively correlated with the probability of conducting an additional study, then (because *ω*_1_ − 1 is negative), the combined estimator ${\hat{\theta }}_{\left(2\right)}$ will be negatively biased.

A somewhat simpler version of our Equation (1) was obtained in equation (2.3) of Ellis and Stewart (2009) who considered equal weights and $p\left({\hat{\theta }}_{1},s_{1}^{2},\theta_{0}\right)=p\left({\hat{\theta }}_{1}>cs_{1}^{2}\right)$, i.e. the second trial is carried out only if the result of the first trial is significant. This decision procedure is not intuitive and was labelled a ‘toy story’ by the authors.

Next, we study the conditional biases given the decision about the next trial. In practice, conditional biases are arguably more important than their unconditional counterparts. When a practitioner or a meta‐analyst finds several trials in the literature, a particular decision‐making scenario may have already taken place. Therefore, a standard meta‐analytic estimate is, in fact, a conditional estimate.

Assume that $E\left(p\left({\hat{\theta }}_{1},s_{1}^{2},\theta_{0}\right)\right)\neq 0$ and let *Y* = 1 if the second trial is conducted and zero otherwise. Suppose *Y* and ${\hat{\theta }}_{2}$ are conditionally independent given $\left({\hat{\theta }}_{1},s_{1}^{2}\right)$. Then the conditional expectation given that the second trial is conducted is

(2) $$E\left\{{\hat{\theta }}_{\left(2\right)}|Y=1\right\}=\theta +\frac{\omega_{1}Cov\left(p\left({\hat{\theta }}_{1},s_{1}^{2},\theta_{0}\right),{\hat{\theta }}_{1}\right)}{E\left(p\left({\hat{\theta }}_{1},s_{1}^{2},\theta_{0}\right)\right)}+\omega_{1}\left\{E\left({\hat{\theta }}_{1}\right)-\theta \right\}\text{.}$$

Thus, unless $Cov\left(p\left({\hat{\theta }}_{1},s_{1}^{2},\theta_{0}\right),{\hat{\theta }}_{1}\right)=0$, both the unconditional and conditional expectations are biased. The last term in the preceding equation, although zero for an unbiased estimator ${\hat{\theta }}_{1}$, is retained intentionally, so that the equation can be generalised to the case of *K* sequential decisions and trials. Similarly, the conditional expectation given that the trial is not conducted is

(3) $$E\left\{{\hat{\theta }}_{\left(2\right)}|Y=0\right\}=\theta -\frac{Cov\left(p\left({\hat{\theta }}_{1},s_{1}^{2},\theta_{0}\right),{\hat{\theta }}_{1}\right)}{1-E\left(p\left({\hat{\theta }}_{1},s_{1}^{2},\theta_{0}\right)\right)}\text{.}$$

The consequence of Equations (2) and (3) is that when the probability of conducting an additional study is positively correlated with the value of the current estimate ${\hat{\theta }}_{1}$, the bias given that the study is conducted is positive, and negative given that it is not.

Remark 1 If *K* trials were run sequentially and the decision to run trial *i* + 1 was dependent on the cumulative results from the first *i* trials, ${\hat{\theta }}_{\left(i\right)}=\sum_{j=1}^{i}\;\omega_{j}{\hat{\theta }}_{j}$ for *i* = 1, ···, *K* − 1. Equation (2) can be applied directly to cumulative effect ${\hat{\theta }}_{\left(K-1\right)}$ and the effect in the *K*‐th trial ${\hat{\theta }}_{K}$, to obtain a recurrent equation for the sequential decision bias

(4) $$E_{K}\left({\hat{\theta }}_{\left(K\right)}-\theta \right)=\omega_{\left(K-1\right)}E_{K-1}\left({\hat{\theta }}_{\left(K-1\right)}-\theta \right)+\omega_{\left(K-1\right)}Cov\left(p_{\left(K-1\right)},{\hat{\theta }}_{\left(K-1\right)}\right){\left[E\left(p_{\left(K-1\right)}\right)\right]}^{-1}\text{.}$$

In Equation (4), E_*i*_(⋅) is the conditional expectation given *i* trials, and $\omega_{\left(i\right)}=\sum_{1}^{i}\;\omega_{j}/\sum_{1}^{i+1}\;\omega_{j}$ is the normalised weight for ${\hat{\theta }}_{\left(i\right)}$. Similarly, $p_{\left(i-1\right)}=p\left({\hat{\theta }}_{\left(i-1\right)},s_{\left(i-1\right)}^{2},\theta_{0}\right)$ is the probability of running the *i*th trial.

### 2.2 Modelling the probability of the second trial

To examine the resulting biases numerically, a model for the probability of running the next trial $p\left(\hat{\theta },s^{2},\theta_{0}\right)$ given cumulative results $\left(\hat{\theta },s^{2}\right)$ is required. We first examine three simple models: the power‐law, the extreme value and the probit models, and then a more complex model depending on power calculations.

### 2.2.1 A power‐law model for $p\left(\hat{\theta },s^{2},\theta_{0}\right)$

**Figure 1:**
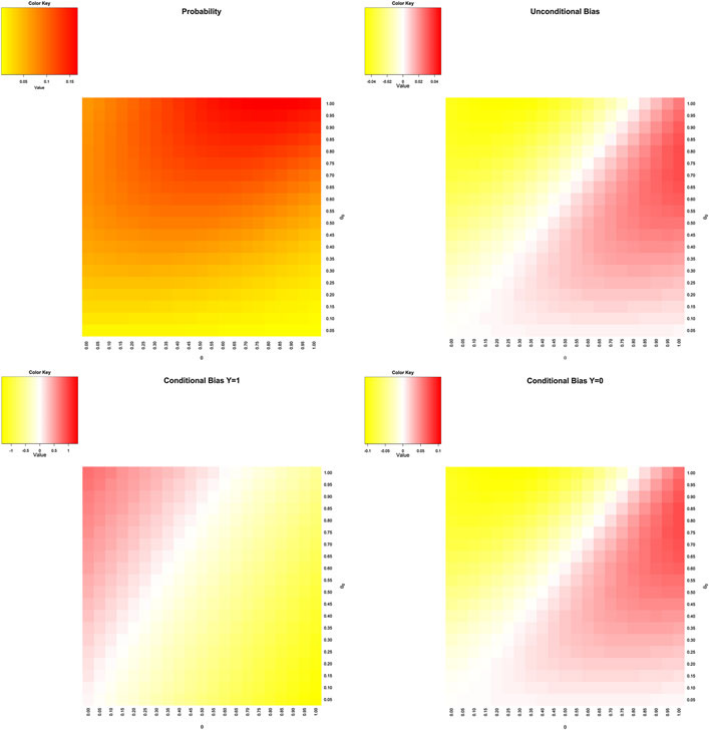
The probability of conducting a second trial and the bias in the unconditional and conditional means when the second trial is conducted (Y = 1) and not conducted (Y = 0), given by Equations (1), (2) and (3), respectively, for the power‐law model of Section 2.2.1 with *t* = 3. The *x*‐axis is the true value of *θ* whilst the *y*‐axis is the target value *θ*. The parameter values are *σ* ^2^ = 1/3 and *ω*_1_ = *ω*_2_ = 1/2.

Suppose ${\hat{\theta }}_{1}∼N\left(\theta ,\sigma^{2}\right)$ for *θ* > 0, and, for some *t* > 0, $p\left({\hat{\theta }}_{1},s^{2},\theta_{0}\right)={\left({\hat{\theta }}_{1}/\theta_{0}\right)}^{t}$ for $0<{\hat{\theta }}_{1}<\theta_{0}$ and zero otherwise. That is, there is no need for further trials when the effect is at least *θ*_0_, and ‘promising’ results increase the probability of the next trial. The variability of the estimator is not taken into account in this very simplistic model. The function *p*(*x*, *θ*_0_) is a distribution function from the power‐law family of distributions on [0, *θ*_0_) and *t* = 1 corresponds to uniform distribution. In (15) in the Supporting Information, we see that the covariance between ${\hat{\theta }}_{1}$ and $p\left({\hat{\theta }}_{1},\theta_{0}\right)$ is negative if *θ* > *θ*_0_, so we expect a negative bias for the conditional expectation in this case. If *θ*_0_ > *θ*, the bias may be positive. In Figure 1, we plot the heatmaps for the biases of the conditional and unconditional means as functions of 0 ≤ *θ*, *θ*_0_ ≤ 1 for *t* = 3, *ω*_1_ = *ω*_2_ = 1/2 and *σ* ^2^ = 1/3 (the value obtained as $s_{1}^{2}/n_{1}$ for the first trial in the example in Section 4). These heatmaps were computed by performing 10 000 simulations at each pair of values of *θ*, *θ*_0_ in steps of 0.05. We note that the most biased estimator is the conditional estimator when a new trial is conducted. It can be seen that in this model, the unconditional mean is reasonably precise, but the step 2 conditional mean has a considerable positive bias when the actual effect is small in comparison with the target value of *θ*_0_. The step 3 conditional mean appears to be even more biased for small values of the actual effect. Because the model assumes no need for a further trial when the effect value of *θ*_0_ is reached, the model underestimates the mean for large values of *a*.

**Figure 2:**
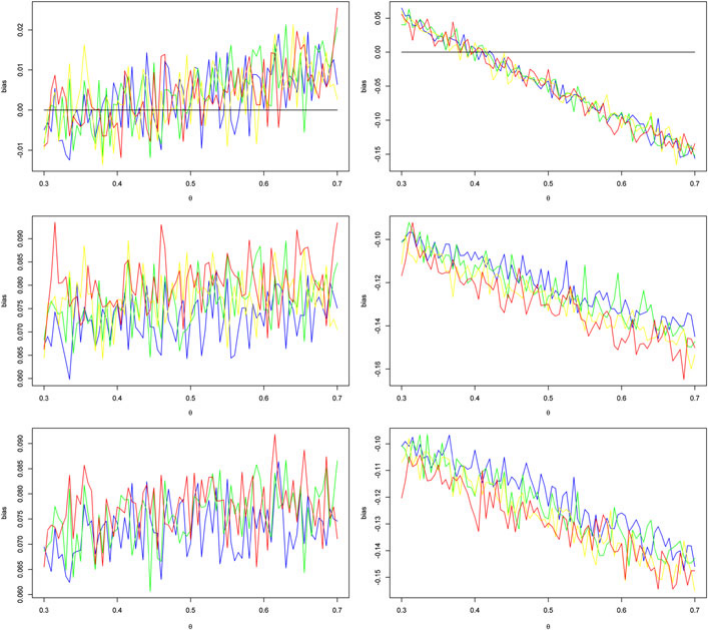
Biases of unconditional (left) and conditional *Y* = 1 (right) expected values of the cumulative effects ${\hat{\theta }}_{\left(2\right)}$ at the second study for *τ* ^2^ values of 0 (blue), 0.02 (green), 0.04 (yellow) and 0.06 (red). Rows 1 to 3 correspond to the biases in power‐law with *t* = 3, extreme value (*r* = 0.8) and probit (*r* = 0.8) with *α* = 0 and *β* = 1 models, respectively. Results from 10 000 simulations at each value of *θ* = 0.3(0.05)0.7 for the target value of *θ*_0_ = 0.5, equal weights *ω*_1_ = *ω*_2_ and the variance *σ* ^2^ = 1/3 (corresponding to the within‐study variance $s_{1}^{2}=19.94$ and sample size of *n*_1_ = 61 in the example of Section 4).

Figure 2 illustrates the biases arising in the random‐effects model ${\hat{\theta }}_{1}∼N\left(\theta ,\sigma^{2}+\tau^{2}\right)$ for 0.3 ≤ *θ* ≤ 0.7 and the target value of *θ*_0_ = 0.5. There is much random variation in this figure due to the comparatively large variance of *σ* ^2^ = 1/3, but the biases are quite distinct. Plots (a) and (b) in this figure show that increases in *τ* ^2^ cause increased biases in the power‐law model. Similar bias plots for the third trial (*i* = 3) following on from the second in the same fashion are given in the Supporting Information, Fig. S1, plots (a) and (b). Biases for *σ* ^2^ = 0.04 (corresponding to a sample size of 500) are given in Figs. S2 and S3 in the Supporting Information. Here, the impact of an increase in *τ* ^2^ is visible very clearly.

#### 2.2.2 Extreme‐value and probit models

In this subsection, we briefly consider two alternative models for $p\left(\hat{\theta },s^{2},\theta_{0}\right)$. Both are based on a class of *t*‐models for publication bias by Copas (2013). These models are of the form *a*(*θ*/*σ*) for an arbitrary function *a*(⋅). We centre these functions at the clinically significant effect *θ*_0_ and truncate at *rθ*_0_ for some 0 < *r* < *θ*_0_, so that the next trial is unlikely when the current estimated effect is much below (or much above) the clinically significant effect. The general form of these models is

$$p\left(\theta ,\sigma^{2},\theta_{0},r\right)=\left[1-G\left(\left(\theta -\theta_{0}\right)/\sigma \right)\right]/\left[1-G\left(\left(r-1\right)\theta_{0}/\sigma \right)\right]\;for\;\theta >r\theta_{0}\text{,}$$

and 0 otherwise, for a distribution function *G*(⋅). Consider first an extreme value distribution‐based model for which the distribution function *G*(*θ*, *σ* ^2^, *θ*_0_) = exp(−exp((*θ*_0_ − *θ*)/*σ*)). The simulation‐based bias plots for the second trial under this model with *r* = 0.8 are given in plots (c) and (d) of Figure 2. It can be seen that the unconditional expected values underestimate the mean, and conditional (*Y* = 1) expected values overestimate the mean. Biases noticeably increase with the increase in the heterogeneity parameter *τ* ^2^.

Following a probit model for publication bias by Copas (2013) we consider the probit model with *G*(*θ*, *σ* ^2^, *θ*_0_) = *Φ*(*α* + *β*(*θ* − *θ*_0_)/*σ*). We provide simulation results for a simple version of this model with *α* = 0 and *β* = 1 for *r* = 0.8 in the plots (e) and (f) of Figure 2. As with the extreme value model, the probit model also yields negative unconditional bias and positive conditional bias given that the next trial is conducted. Once more, the biases increase with an increase in the heterogeneity parameter *τ* ^2^.

The bias plots for the third trial (*i* = 3) are given in the Supporting Information, Fig. S1. It can be seen that the biases increase with each step *i* in decision‐making.

As we have seen, different rules and different parameters could give quite different results, but these indicate that biases do occur when data‐dependent rules are used to determine if the second trial should be conducted.

#### 2.2.3 A power calculation model for $p\left({\hat{\theta }}_{1},s_{1}^{2},\theta_{0}\right)$

In this section, we look at the situation in which the probability of conducting a new trial may depend on power calculations. For simplicity, suppose that two studies may be conducted and that we use normally distributed means to estimate an effect size *θ* that is the same for each trial. Typically, if the power calculations yield a small sample size for the second study, the increase in total power of the subsequent meta‐analysis will be minor, and it may be decided that it is not worth proceeding with the study. Alternatively, the power calculations may yield a large sample size and it may not be possible to achieve the desired power with the available resources.

Let the first study result in an estimate ${\hat{\theta }}_{1}$ of *θ*. We wish to determine a sample size *n*_2_ for the next study, so that the combined effect ${\hat{\theta }}_{\left(2\right)}=\left(w_{1}{\hat{\theta }}_{1}+w_{2}{\hat{\theta }}_{2}\right)/\left(w_{1}+w_{2}\right)$ will be significantly different from zero (two‐sided) at the significance level *α* with 1 − *β* power at the target effect size *θ*_0_. Here, $w_{i}=n_{i}/\sigma_{i}^{2}$, *i* = 1, 2 are the unnormalised inverse variance weights, and $\sigma_{i}^{2}$ are the population variances within the studies. The level *α* should be chosen to account for multiple testing, but the details of such adjustments are beyond the scope of this paper. The variance of the combined effect is then (*w*_1_ + *w*_2_)^− 1^. In the Supporting Information, we show that the required sample size is

(5) $$n_{2}=\left(\frac{c^{2}}{\theta_{0}^{2}}-w_{1}\right)\sigma_{2}^{2}\text{,}$$

where *c* = *z*_1 − *α*/2_ + *z*_1 − *β*_.

In the absence of clinical knowledge, the estimate of the treatment effect from the initial study is often taken to be the clinically relevant treatment effect for this sample size calculation – a practice we do not condone. In this scenario, the second sample size is calculated using the estimated mean ${\hat{\theta }}_{1}$ from the first trial as the effect size *θ*_0_. The estimated variance $s_{1}^{2}$ may be used to estimate both $\sigma_{1}^{2}$ and $\sigma_{2}^{2}$ in the aforementioned formula. Then the sample size is taken to be

(6) $$n_{2}=\frac{c^{2}s_{1}^{2}}{{\hat{\theta }}_{1}^{2}}-n_{1}\text{.}$$

If ${\hat{\theta }}_{1}$ is normally distributed and independent of the sample variance $s_{1}^{2}$, which has *d*_1_ degrees of freedom (*d*_1_ = *n*_1_ − 1 for one sample, but we introduce this notation for more generality), then $d_{1}s_{1}^{2}/\sigma_{1}^{2}∼\chi^{2}\left(d_{1}\right)$, and we may compute probabilities associated with the experiment. For example, suppose that it is decided to conduct a new study of size *b* if *a* < *n*_2_ ≤ *b* for some *a* and *b*. Then assuming that ${\hat{\theta }}_{1}∼N\left(\theta ,\sigma_{1}^{2}/n_{1}\right)$ is unbiased, the conditional (given ${\hat{\theta }}_{1}=\theta_{1}$) and unconditional probabilities that a new trial is conducted are given by equations (18) and (19) in the Supporting Information Section A.

**Figure 3:**
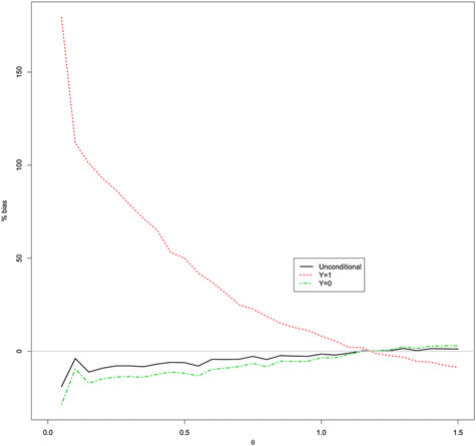
Estimated percent unconditional (solid line) and conditional (Y = 1 dashed line, Y = 0 dot‐dashed line) bias from 10 000 simulations as a function of *θ* for the power calculation rule of Section 2.2.3, with $\sigma_{1}^{2}=20$, *n*_1_ = 60, *a* = 30 and *b* = 80 and a second trial of size 80 is conducted if *a* < *n*_2_ < *b*.

In Figure 3, we plot the estimated percentage unconditional bias as a function of *θ* from 10 000 simulated experiments for the same scenario with *n*_1_ = 60, $\sigma_{1}^{2}=20$ and if a second experiment of size 80 is conducted when 30 ≤ *n*_2_ ≤ 80. The conditional bias is calculated over the simulations where a second trial was/was not conducted. The biases are very considerable, especially for small effects *θ*, where the conditional bias given that a new trial is conducted is above 100 %, and the negative unconditional bias is approximately 10 %.

In Fig. S4 in the Supporting Information, we plot the conditional and unconditional probabilities that a new trial is conducted for *n*_1_ = 60, and $\sigma_{1}^{2}=20$, the parameters taken from the first trial in the Example discussed in Section 4. We assume *a* = 30 and *b* = 80 in these plots.

Remark 2 If the decision to run trial *i* + 1 of size *b* is taken if *a* < *n*_*i* + 1_ ≤ *b* for some *i* ≥ 1, denote the cumulative combined effect from the first *i* trials ${\hat{\theta }}_{\left(i\right)}=\sum_{j=1}^{i}\;w_{j}{\hat{\theta }}_{j}/W_{\left(i\right)}$ for $W_{\left(i\right)}=\sum_{j=1}^{i}\;w_{j}$, and the cumulative sample size $n_{\left(i\right)}=\sum_{j=1}^{i}\;n_{j}$. Equation (5) changes to

(7) $$n_{i+1}=\left(\frac{c^{2}}{\theta_{0}^{2}}-W_{\left(i\right)}\right)\sigma_{i+1}^{2}\text{,}$$

where $\sigma_{i+1}^{2}$ is the variance of the trial (*i* + 1). Equations (18) and (19) in the Supporting Information Section A can be adapted to provide the conditional and unconditional probabilities of the trial (*i* + 1) being conducted.

## 3 Sequential design bias

Although there is an implicit decision made prior to designing the second trial, we now emphasise the design of the second trial and refer to the resulting bias as design bias. We continue the approach and the notation of Section 3, but now rather than conduct a new experiment with *b* observations, we conduct it with *n*_2_ observations. We investigate the bias introduced by sample size calculations based on estimated effects and their variances. For the *i*th trial, *i* = 1, 2, with *n*_*i*_ observations, the weight is $w_{i}=n_{i}/\sigma_{i}^{2}$ and the effect estimate ${\hat{\theta }}_{i}$ is taken to be a sample mean. The combined estimate over two trials is then ${\hat{\theta }}_{\left(2\right)}=\sum_{i=1}^{2}\;w_{i}{\hat{\theta }}_{i}/\sum_{i=1}^{2}\;w_{i}$. Note that *n*_2_ = 0 yields *w*_2_ = 0 and ${\hat{\theta }}_{\left(2\right)}={\hat{\theta }}_{1}$. In what follows, we make the assumption that the estimates of the effect size and variances are independent, which will hold for samples from normal populations and approximately for other situations.

In practice, $\sigma_{2}^{2}$ is not known and we must guess a value to determine the sample size. Denote this by $\sigma_{g}^{2}$. A common option is to take $\sigma_{g}^{2}={\hat{\sigma }}_{1}^{2}$, which is explored later. Then, following from Equation (5), we take

$$n_{2}=\left(\frac{c^{2}}{\theta_{0}^{2}}-w_{1}\right)\sigma_{g}^{2}=\left(\frac{c^{2}}{\theta_{0}^{2}}-w_{1}\right)d^{2}\sigma_{2}^{2},$$

where $d^{2}=\sigma_{g}^{2}/\sigma_{2}^{2}$. Thus, *n*_2_ is positive if $c^{2}>w_{1}\theta_{0}^{2}$ or $n_{1}<c^{2}\sigma_{1}^{2}/\theta_{0}^{2}$. Let *n*_2_ = max(*n*_2_, 0). Set *d* = 0 whenever *n*_2_ = 0. Therefore, with $w_{2}=n_{2}/\sigma_{2}^{2}=\left(c^{2}/\theta_{0}^{2}-w_{1}\right)d^{2}$, we have

(8) $${\hat{\theta }}_{\left(2\right)}=\frac{w_{1}{\hat{\theta }}_{1}+w_{2}{\hat{\theta }}_{2}}{w_{1}+w_{2}}={\hat{\theta }}_{2}+\frac{w_{1}\left({\hat{\theta }}_{1}-{\hat{\theta }}_{2}\right)}{w_{1}+w_{2}}\text{.}$$

As long as the value of *θ*_0_ used in the sample size calculation is a constant decided by clinical considerations, the expected value of the cumulative effect given by Equation (8) is equal to *θ* and is unbiased. But in the absence of this clinical knowledge, when designing the second study, it is tempting to use the value of ${\hat{\theta }}_{1}+\delta$ for some constant *δ* for the sample size calculation. In clinical trials, this can form the basis of proceeding to a phase III trial. That is, we now have

(9) $$n_{2}=\left(\frac{c^{2}}{{\left({\hat{\theta }}_{1}+\delta \right)}^{2}}-w_{1}\right)\sigma_{g}^{2}$$

and ${\hat{w}}_{2}=\left(c^{2}/{\left({\hat{\theta }}_{1}+\delta \right)}^{2}-w_{1}\right)d^{2}$. To examine this situation, for simplicity, suppose $\sigma_{1}^{2}$ and therefore *w*_1_ too are known. Note that if ${\hat{\theta }}_{1}$ is large, then *n*_2_ given by (9) can be negative, and in this case, a further experiment is not conducted.

In the Supporting Information Section A, we show that if we stop after the first experiment because the observed result had the desired power for the observed effect size, we can obtain a highly positively biased estimate of *θ*. For example, if *θ* = 0.2, $\sigma_{1}^{2}=1,$ *α* = 0.05, *β* = 0.2, *δ* = 0.2 and *n*_1_ = 15, then $E\left({\hat{\theta }}_{1}|n_{2}\leq 0\right)=0.647>>0.2=\theta$. Fortunately, the bias diminishes with increase in *n*_1_, so that for *n*_1_ = 50, $E\left({\hat{\theta }}_{1}|n_{2}\leq 0\right)=0.310$, and for *n*_1_ = 100, $E\left({\hat{\theta }}_{1}|n_{2}\leq 0\right)=0.222$. In the limit, the bias is zero. Similarly, given that the observed result has not reached the desired power and the second trial is conducted, the estimate of *θ* is negatively biased.

Now let us explore the unconditional bias of *θ*. Suppose that we guess the variance $\sigma_{2}^{2}$ exactly, i.e. *d* = 1. As a consequence of (20) in the Supporting Information Section A, we have

(10) $$E\left({\hat{\theta }}_{\left(2\right)}\right)\leq \theta +\left(\frac{\phi \left(h\right)\sigma_{1}}{\sqrt{n_{1}}}+\frac{2\left(\theta +\delta \right)}{c^{2}}\right)\text{,}$$

where $h=\left\{\left(\sqrt{c^{2}/w_{1}}-\delta \right)-\theta \right\}/\left(\sigma_{1}/\sqrt{n_{1}}\right)$ and *ϕ*(⋅) is the standard normal density, giving an upper bound on the unconditional bias of the estimate ${\hat{\theta }}_{\left(2\right)}$. That is, if *δ* > 0 and *α* = 0.05, *β* = 0.2 then *c* ^2^ = 7.85 and the bias is not greater than 25%. But what happens when *d* ≠ 1?

In the general case, for an arbitrary *d* value,

$${\hat{\theta }}_{\left(2\right)}={\hat{\theta }}_{2}+\frac{w_{1}\left({\hat{\theta }}_{1}-{\hat{\theta }}_{2}\right){\left({\hat{\theta }}_{1}+\delta \right)}^{2}}{w_{1}{\left({\hat{\theta }}_{1}+\delta \right)}^{2}\left(1-d^{2}\right)+d^{2}c^{2}}\text{.}$$

At *d* = 0, this is just ${\hat{\theta }}_{1}$ and is unbiased. However, this is of little practical use for at *d* = 0 we would not conduct the second experiment. Now, for an arbitrary *d*,

(11) $$E\left({\hat{\theta }}_{\left(2\right)}\right)=\theta +E\left\{\frac{w_{1}\left({\hat{\theta }}_{1}-\theta \right){\left({\hat{\theta }}_{1}+\delta \right)}^{2}}{w_{1}{\left({\hat{\theta }}_{1}+\delta \right)}^{2}\left(1-d^{2}\right)+d^{2}c^{2}}\right\}\text{,}$$

which is analytically intractable.

We have seen that the bias at *d* = 1 can be quite large; therefore, we conducted a series of simulations to examine the bias for other values of *d*. The design of these simulations is described in the Supporting Information Section C.

**Figure 4:**
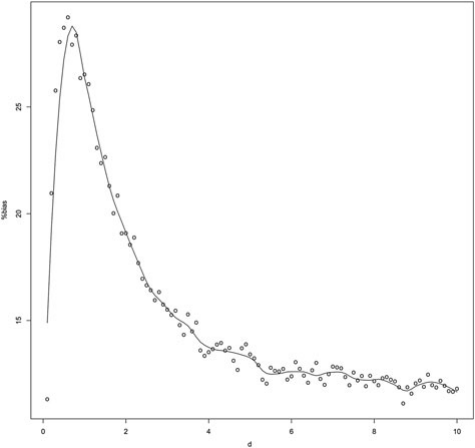
Plot of the percent bias against *d* from 10 000 simulations with *θ* = 0.2, *δ* = 0.2, *α* = 0.05 and *β* = 0.2. The variances $\sigma_{1}^{2}=19.94$, $\sigma_{2}^{2}=24.96$ and the sample size *n*_1_ = 61 were taken from the example of a meta‐analysis discussed in Section 4. The locfit (Loader, 2012) package was used to smoothly estimate the mean bias.

For our first simulations, we took *θ* = 0.2, *δ* = 0.2, *α* = 0.05 and *β* = 0.2. The variances $\sigma_{1}^{2}=19.94$, $\sigma_{2}^{2}=24.96$ and the sample size *n*_1_ = 61 were taken from the example of a meta‐analysis discussed in Section 4 so that *w*_1_ = 3.06. Then *c* ^2^/*θ* ^2^ − *w*_1_ = 136.22 > 0. We conducted 10 000 simulated initial experiments and took *d* from 0.1 to 10 in steps of 0.1. The means of the combined estimators for each value of *d* are plotted in Figure 4. As there is some variability due to the random sampling, we used the R (R Core Team, 2012) package locfit (Loader, 2012) to smoothly estimate the mean. It is clear from this plot that the bias can be substantial over a range of guesses for $\sigma_{2}^{2}$. With *δ* = 0, the bias was around 15% less than that in Figure 4 but still of concern. If we took $\sigma_{g}^{2}={\hat{\sigma }}_{1}^{2}$, then the mean percentage bias over the simulations was 49.9% with standard deviation of 3.16 so the bias was uniformly high. As a check, if we used *θ* and $\sigma_{2}^{2}$ with *δ* = 0 to compute *n*_2_, then the mean bias was close to zero (0.129%). The bias was 0.042%, also close to zero if we used *θ* and ${\hat{\sigma }}_{1}^{2}$ again with *δ* = 0 to compute *n*_2_, which confirms that the bias arises from using the estimated value of ${\hat{\theta }}_{1}$ to decide to carry out the second experiment and compute the sample size.

## 4 Example

**Figure 5:**
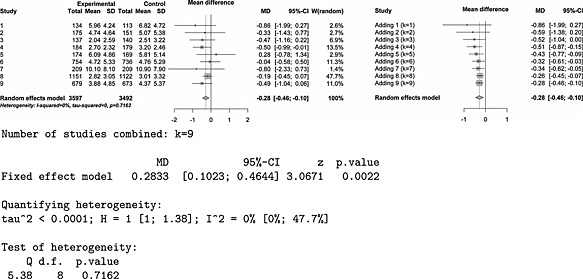
Forest plot (left), cumulative meta‐analysis plot (right) and some additional results for Johnson(1993) meta‐analysis.

The meta‐analysis conducted by Johnson (1993) comprised nine studies comparing sodium monofluorophosphate with sodium fluoride dentifrices in the prevention of caries development. The outcome of interest was the dental decay score, and a summary of the data, the forest plot, the cumulative meta‐analysis plot and the results obtained with R package ‘meta’ (Schwarzer, 2010) are given in Figure 5. Heterogeneity was not detected, so the fixed‐effect model was used. The first three studies in this meta‐analysis failed to reach significance but showed positive effect. The combined effect after three trials, ${\hat{\theta }}_{\left(3\right)}=0.5211$, is just significant (*p* = 0.049, confidence interval [0.003; 1.039]).

For purposes of an illustrative example, we will assume the following: that the meta‐analyst could have been responsible first for deciding whether or not to conduct the second (and/or third) study given the results of the previous studies and second for deciding on the design of the additional study. We will examine the bias that would be introduced by these decisions.

All results formulated in the previous sections were based on just one sample size *n*_*i*_ and the sample variance $s_{i}^{2}$ per trial. Here, we first explain how to apply them to the standard clinical trial setting. Consider *K* comparative studies with the treatment (*T*) and the control (*C*) arms of sample sizes *N*_*iT*_ and *N*_*iC*_, *N*_*iT*_ + *N*_*iC*_ = *N*_*i*_. The effect measure is the mean difference ${\hat{\theta }}_{i}={\bar{X}}_{\text{iT}}-{\bar{X}}_{iC}$. Assume, for simplicity, equal variances $\sigma_{i}^{2}$ in both arms of each study. The variance of the effect is $Var\left({\hat{\theta }}_{i}\right)=\sigma_{i}^{2}\left(N_{iC}^{-1}+N_{\text{iT}}^{-1}\right)$. This can be written as $\sigma_{i}^{2}/n_{i}$ for the effective sample size *n*_*i*_. This effective sample size is the geometric mean of the sample sizes of each arm, i.e. $n_{i}={\left(N_{\text{iT}}^{-1}+N_{iC}^{-1}\right)}^{-1}$. For a balanced trial of size *N*_*i*_, *n*_*i*_ = *N*_*i*_/4. Now all the theory of Sections 2 and 3 can be applied using effects ${\hat{\theta }}_{i}$, effective sample sizes *n*_*i*_ and pooled sample variances $s_{i}^{2}$ with *d*_*i*_ = *N*_*i*_ − 2 d.f. from each study.

For the first three trials, the effective sample sizes were *n* = (61.30, 81.06, 69.24) and the pooled sample variances were *s* ^2^ = (19.94, 24.96, 8.56), respectively. Assuming standard choices of a 5% significance level and power of 80%, the constant *c* in Equations (6) and (9) is *c* = *z*_1 − *α*/2_ + *z*_1 − *β*_ = 2.802.

After the first trial, we consider performing the second trial with a goal of achieving a definitive meta‐analysis. Suppose the second trial may run given that its sample size is between 200 and 2000 patients. This translates into the decision to conduct a new balanced trial if *a* < *n*_2_ ≤ *b* for *a* = 50 and *b* = 500. Using the estimated treatment effect ${\hat{\theta }}_{1}=0.86$, we calculate from (6) the value of *n*_2_ = 150.35 required for this new trial. Assume that the true parameter values are *θ* = 0.28 (the combined effect from nine trials) and *σ* ^2^ = 21.62 (the pooled variance from nine trials). Under these assumptions, we estimate from equation (19) in the Supporting Information the unconditional probability of continuation after trial 1 to be *p* = 0.349. If we do not wish to restrict sample sizes from below, take *a* = 0, and the resulting estimated probability of continuation is *p* = 0.412. To assess the resulting bias after two trials, we performed 10 000 simulated experiments with *n*_1_ = 61 (effective sample size of the first trial), *θ* = 0.28 and *σ* ^2^ = 21.62 for *a* = 50 and *b* = 500. From the simulations, the estimated probability to continue after the first trial is 0.343 (in agreement with our theoretical estimate of 0.349), unconditional bias after two trials is − 19.92 %, conditional bias given the decision to stop is − 34.40 % and conditional bias given the decision to continue is 8.13 %. Thus, if the meta‐analyst had been responsible for deciding (based on the results of the first study) whether to conduct the second study, then a substantial sequential decision bias would have been introduced.

If the second trial was run first, the estimated effective sample size for the next trial would be 1719 > *b*, and the next trial would not be run. Now suppose that the first two trials were run independently from each other, but the decision is required about the third trial. The variances in these two trials are similar, so we proceed as suggested in Remark 2. The required sample size calculated from (7) is 376.02. The unconditional probability to continue is 0.25. Taking *a* = 0 increases this probability to 0.30. As it happened, the third trial was run with an effective sample size of 69.24, resulting in marginal significance of the combined effect ${\hat{\theta }}_{\left(3\right)}$ of sodium monofluorophosphate.

So far, we considered the sequential decision scenario for this meta‐analysis. To assess the sequential design bias in this realistic setting, suppose that after the first trial the investigators correctly assume that the effect is overestimated and use *δ* = − 0.36 to ‘correct’ the effect to 0.50 in the sample size calculation. We have performed 10 000 simulated experiments with *n*_1_ = 61, *θ* = 0.28, $\sigma_{1}^{2}=19.94$, $\sigma_{2}^{2}=24.96$ and *δ* = − 0.36. The results are plotted in Fig. S5 in the Supporting Information Section C. For the values of *d* ≈ 1, i.e. when the variance is guessed correctly, the bias is about 15 %. The bias is greatly reduced for the large values of *d*. This is to be expected as the large assumed variance would result in large sample size of the next trial.

It is clear that in this meta‐analysis with moderate sample sizes, both types of biases are far from negligible.

## 5 Discussion

We have demonstrated theoretically and by simulations that both sequential decision bias and sequential design bias can arise in sequential and cumulative meta‐analyses when the results of previous studies influence the design of a new study. This setting differs from the standard sequential meta‐analysis setting in that a meta‐analyst has an active role in the design of the subsequent trial aiming at a definitive meta‐analysis. We have seen that both the conditional and unconditional biases can be non‐negligible. Thus, caution needs to be exercised in conducting meta‐analysis when prior knowledge has been used to design the trials being studied.

Our sample size calculations are based on the unconditional power of the Wald test for the combined effect. A recent paper by Roloff *et al*. (2013) advocated using the conditional power approach. We do not expect this to alleviate the bias. The design bias arising from the conditional power is discussed in the designed extension of a clinical trial setting by Denne (2000). In particular, that paper compared the biases of the estimated effects in conditional and unconditional setting and found that the differences were minor (see Figure 2 in Denne (2000)).

We considered both the fixed‐effect and the random‐effects models of meta‐analysis and demonstrated analytically and by simulations that in both settings the problems due to sequential biases are apparent. According to our simulations, the sequential biases increase with increased heterogeneity.

Sequential meta‐analysis results in inflation of type‐I error due to multiple testing. This well‐known issue was not discussed in any detail in this paper. A number of procedures aimed at adjustment of significance levels to safeguard the overall type‐I error are available from a number of publications such as Pogue and Yusuf (1998), van der Tweel and Bollen (2010) and Higgins *et al*. (2011). Such adjustments will result in larger sample sizes of the new studies (formulae (6) and (9)) and may decrease sequential biases (formula (11)) through increases in critical values. Bias due to non‐independence of studies as well as the timing of the meta‐analysis itself was studied theoretically but without quantification by Ellis and Stewart (2009). Their findings are akin to the sequential decision bias discussed in Section 2.

Our models assumed that a new study is more likely if the existing evidence is in favour of a new treatment than if it is the other way around. However, we have not considered in detail how the interplay between the effect size and the uncertainty may affect the sequential biases. The value of information approach (Claxton & Sculpher, 2006; Claxton *et al*., 2002) is an alternative method to decide on the necessity of further research. This method is based on economic modelling comparing the costs involved in further research with benefits of reduction in uncertainty. This method is widely used in contemporary health policy decision‐making (Claxton & Sculpher, 2006). It would be of great interest to investigate the existence of sequential decision bias resulting from this approach.

Note that in clinical trials, the favourable results of a phase II trial may be used to design the phase III trial, ‘*Estimates of treatment effects and variability from earlier trials are traditionally used in the design of trials at the next stage*’ (Kirby *et al*., 2012). This setting is different from meta‐analysis in that results are not combined. Moreover, the decision to conduct the phase III trial depends on a significant result in the phase II trial, whereas with meta‐analysis guiding research, the sequence of trials may be terminated once significance is attained. However, the problem of resulting biases is already recognised in drug development, (Wang *et al*., 2006) and methods of adjustment are sought (Kirby *et al*., 2012). Perhaps a closer analogy for the sequential decision bias is with group‐sequential clinical trials, where a significant result at an interim stage would stop the trial, but otherwise, the results of sequential interim stages are accumulated and combined. In this setting, the existence of sequential bias is widely recognised and the means of adjustment for this bias have been developed (Whitehead, 1986). This adjustment is possible because of the explicit decision rules in these trials. Design bias is similar to the bias induced by mid‐trial sample size re‐estimation in adaptive trials (Li *et al*., 2002; Wang *et al*., 2010). However, methods of sequential bias adjustment in meta‐analytic setting are more difficult to develop than in sequential and adaptive clinical trials. The bias depends not only on the unknown true value or the precision of the effect *θ* but also on the strategy for making the decision to continue or stop and of choosing the sample size of the next study. If such a strategy is made explicit, by, say, the Research Councils, development of an appropriate bias adjustment should be possible. Development of such a strategy appears to be an important and complicated problem deserving concerted efforts of statisticians and decision‐makers.

As was pointed out by the Associate Editor, there are clear parallels between the models for the sequential decision bias and the models for publication bias, although the decisions being made are aimed at the next trial or at the current trial, respectively. In fact, both the model by Ellis and Stewart (2009) and our extreme‐value and probit models are the publication bias models from the *t*‐family introduced by Copas (2013). Similar to publication bias models, the choice of the model may greatly affect the amount of bias, but we have demonstrated that non‐negligible biases may arise in a variety of models, and we recommend the use of multiple models for sensitivity analyses to uncover the consequences of sequential biases. To enable this use, all our R programmes are provided in the Supplementary Information materials.

We believe that this is the first time that this important issue is raised in the context of the sequential decision‐making associated with the managed accumulation of evidence. Existence of sequential biases raises a number of important research questions. What is the best way to decide on the usefulness of a new trial? How to design this trial so that the resulting combined estimate is the least biased? How to adjust the combined effect to minimise this bias? All these questions need to be addressed if we are to aim at evidence‐based development of science.

## Supporting information

Supporting info item

Supporting info item

Supporting info item

Supporting info item
