# Supplementary material for: Sequential biases in accumulating evidence
Source: Res Synth Methods. 2015 Dec 1;7(3):294–305. doi: 10.1002/jrsm.1185 (PMC5031232; doi:10.1002/jrsm.1185)
Supplement: Supplementary file 1 — Supporting info item [file JRSM-7-294-s001.pdf]

# Web Appendix

# A Proofs

## Proofs

We give some details of the technical results. To establish (1) we have

$$\begin{aligned} E(\widehat{\theta}_{(2)}) &= E\{p(\hat{\theta}_1,\theta_0)(w_1\hat{\theta}_1 + w_2\hat{\theta}_2) + (1-p(\hat{\theta}_1,\theta_0))\hat{\theta}_1\} \\ &= E\{p(\hat{\theta}_1,\theta_0)w_1\hat{\theta}_1 + p(\hat{\theta}_1,\theta_0)(1-w_1)\hat{\theta}_2 + (1-p(\hat{\theta}_1,\theta_0))\hat{\theta}_1\} \\ &= E[E\{p(\hat{\theta}_1,\theta_0)w_1\hat{\theta}_1 + p(\hat{\theta}_1,\theta_0)(1-w_1)\hat{\theta}_2 + (1-p(\hat{\theta}_1,\theta_0))\hat{\theta}_1|\hat{\theta}_1\}] \\ &= E\{p(\hat{\theta}_1,\theta_0)w_1\hat{\theta}_1 + p(\hat{\theta}_1,\theta_0)(1-w_1)\theta + (1-p(\hat{\theta}_1,\theta_0))\hat{\theta}_1\} \\ &= E\{p(\hat{\theta}_1,\theta_0)(1-w_1)\theta + \hat{\theta}_1 - p(\hat{\theta}_1,\theta_0)(1-w_1)\hat{\theta}_1\} \\ &= \theta + (w_1-1)\mathrm{Cov}(p(\hat{\theta}_1,\theta_0),\hat{\theta}_1). \end{aligned} \quad (12)$$

To establish (2) we have

$$\begin{aligned} E\{\hat{\theta}_{(2)}|Y=1)\} &= \frac{E\{(w_1\hat{\theta}_1 + w_2\hat{\theta}_2)Y\}}{P(Y=1)} \\ &= \frac{E[E\{(w_1\hat{\theta}_1 + w_2\hat{\theta}_2)Y|\hat{\theta}_1\}]}{P(Y=1)} \\ &= \frac{E\{w_1\hat{\theta}_1 p(\hat{\theta}_1,\theta_0) + (1-w_1)\theta p(\hat{\theta}_1,\theta_0)\}}{P(Y=1)} \\ &= \frac{\theta E(p(\hat{\theta}_1,\theta_0)) + w_1(\hat{\theta}_1 p(\hat{\theta}_1,\theta_0) - \theta p(\hat{\theta}_1,\theta_0)}{E(p(\hat{\theta}_1,\theta_0))} \\ &= \theta + \frac{w_1\mathrm{Cov}(p(\hat{\theta}_1,\theta_0),\hat{\theta}_1)}{E(p(\hat{\theta}_1,\theta_0))} + w_1\{E(\hat{\theta}_1)-\theta\}. \end{aligned} \quad (13)$$

To establish (3) note that

$$\begin{aligned} E\{\hat{\theta}_{(2)}|Y=0)\} &= E(\hat{\theta}_1|Y=0) \\ &= \frac{E\{\hat{\theta}_1(1-Y)\}}{P(Y=0)} \\ &= \frac{E\{\hat{\theta}_1(1-E(Y|\hat{\theta}_1))\}}{P(Y=0)} \\ &= \frac{E\{\hat{\theta}_1(1-p(\hat{\theta}_1,\theta_0))\}}{P(Y=0)} \\ &= \theta - \frac{\mathrm{Cov}(p(\hat{\theta}_1,\theta_0),\hat{\theta}_1)}{1-E(p(\hat{\theta}_1,\theta_0))} \end{aligned} \quad (14)$$

The calculations for the power-law model involve the integrals:

$$
\begin{aligned}
E\{p(X,\theta_0)\} &= \int_0^{\theta_0}\left(\frac{x}{\theta_0}\right)^s \frac{1}{\sqrt{2\pi}\sigma}e^{-(x-\theta)^2/(2\sigma^2)}dx \\
E\{Xp(X,\theta_0))\} &= \theta_0\int_0^{\theta_0}\left(\frac{x}{\theta_0}\right)^{s+1} \frac{1}{\sqrt{2\pi}\sigma}e^{-(x-\theta)^2/(2\sigma^2)}dx, \\
\mathrm{Cov}(X,p(X,\theta_0) &= \int_0^{\theta_0}\left(\frac{x}{\theta_0}\right)^s (x-\theta)\frac{1}{\sqrt{2\pi}\sigma}e^{-(x-\theta)^2/(2\sigma^2)}dx.
\end{aligned} \tag{15}
$$

To obtain (5) the sample size calculation for the Wald test of the combined effect in meta-analysis is based on setting

$$
\frac{\theta_0}{\sqrt{\mathrm{var}(\hat\theta_{(2)})}} = \theta_0\sqrt{w_1+w_2} = z_{1-\alpha/2}+z_{1-\beta}. \tag{16}
$$

Now, $w_2 = n_2/\sigma_2^2$ and the resulting equation is solved for $n_2$ yielding

$$
n_2 = \frac{(z_{1-\alpha/2}+z_{1-\beta})^2\sigma_2^2}{\theta_0^2} - n_1\frac{\sigma_2^2}{\sigma_1^2} = \left(\frac{c^2}{\theta_0^2}-w_1\right)\sigma_2^2, \tag{17}
$$

where $c = z_{1-\alpha/2}+z_{1-\beta}$.

In Section 2.2.3, the conditional probability a new trial is conducted given $\hat\theta_1 = \theta_1$, is

$$
\begin{aligned}
P(a,b,\theta_1) &= P(a \le n_2 \le b|\hat\theta_1=\theta_1) \\
&= P\left(a \le \frac{c^2 s_1^2}{\hat\theta_1^2} - n_1 \le b \middle| \hat\theta_1 = \theta_1\right) \\
&= P\left(\frac{(a+n_1)\theta_1^2}{c^2} \le s_1^2 \le \frac{(b+n_1)\theta_1^2}{c^2}\right) \\
&= P\left(\frac{(a+n_1)d_1\theta_1^2}{c^2\sigma_1^2} \le \chi^2(d_1) \le \frac{(a+n_1)d_1\theta_1^2}{c^2\sigma_1^2}\right).
\end{aligned} \tag{18}
$$

The unconditional probability of conducting a new trial as a function of $\theta$ may be computed by integrating the conditional probability over the density $f(\theta_1;\theta,\sigma_1^2)$ of $\theta_1$. If $\hat\theta_1 \sim N(\theta,\sigma_1^2/n_1)$ is unbiased, there is no need to perform this integration explicitly. Instead, note that $F = n_1\hat\theta_1^2/s_1^2$ has a non-central $F_{1,d_1}(\lambda)$ distribution with non-centrality parameter $\lambda = n_1\theta^2/\sigma_1^2$. If a new trial is conducted when $a \le n_2 \le b$, the unconditional probability of this is

$$
P\left\{a < \frac{c^2 n_1}{F} - n_1 \le b\right\} = P\left(\frac{c^2 n_1}{b+n_1} \le F_{1,d_1}(\lambda) \le \frac{c^2 n_1}{a+n_1}\right). \tag{19}
$$

To generalise equations (18) and (19) to trial $i+1$, as mentioned in Remark 2, consider equation (7). The variance of the trial $(i+1)$ in this equation is denoted by $\sigma_{i+1}^2$. If the variances across trials were assumed equal, this variance can be estimated by the pooled sample variance $s_{(i)}^2$. Also, for reasonably large study sizes, $n_{(i)}^* = W_{(i)}s_{(i)}^2 \approx n_{(i)}$. Therefore, under the homogeneity of study variances, the conditional and unconditional probabilities of a new trial being conducted are approximated by equations (18) and (19) with the substitution of $n_{(i)}^*$, degrees of freedom $d_{(i)} = n_{(i)} - i$ and the cumulative combined effect $\hat\theta_{(i)}$ instead of $n_1$, $d_1$ and $\hat\theta_1$, respectively.

For the Example in Section 4, when we consider the 3rd trial after the first two trials, the value of $n_{(2)}^* = 141.27$ is very close to the cumulative effective sample size $n_{(2)} = 142.36$.

In Section 3, the conditional expected value

$$E(\hat{\theta}_1|n_2 \le 0) = E\left(\hat{\theta}_1\middle|\hat{\theta}_1 \ge \sqrt{\frac{c^2}{w_1}} - \delta\right) = \theta + \frac{\sigma_1}{\sqrt{n_1}}\frac{\phi(h)}{1-\Phi(h)}$$

where $h = \{(\sqrt{c^2/w_1} - \delta) - \theta\}/(\sigma_1/\sqrt{n_1})$. For example, if $\theta = 0.2$, $\sigma_1^2 = 1$, $\alpha = 0.05$, $\beta = 0.2$, $\delta = 0.2$ and $n_1 = 15$ then $E(\hat{\theta}_1|n_2 \le 0) = 0.647 >> 0.2 = \theta$. Similarly,

$$E(\hat{\theta}_1|n_2 > 0) = E\left(\hat{\theta}_1\middle|\hat{\theta}_1 < \sqrt{\frac{c^2}{w_1}} - \delta\right) = \theta - \frac{\sigma_1}{\sqrt{n_1}}\frac{\phi(h)}{\Phi(h)}.$$

Then $E(\hat{\theta}_{(2)}) = E(\hat{\theta}_1|n_2 \le 0)\{1 - \Phi(h)\} + E(\hat{\theta}_{(2)}|n_2 > 0)\Phi(h)$.

Finally, to obtain (10) note that if $d = 1$ and noting that $w_1/(w_1 + w_2) = w_1(\hat{\theta}_1 + \delta)^2/c^2$, if $n_2 > 0$ then $\hat{\theta}_{(2)} = \hat{\theta}_2 + (\hat{\theta}_1 - \hat{\theta}_2)(\hat{\theta}_1 + \delta)^2 w_1/c^2$. As $\hat{\theta}_1$ and $\hat{\theta}_2$ are independent and noting $\mathrm{Var}(\hat{\theta}_1) = 1/w_1$, we see that

$$\begin{aligned} E(\hat{\theta}_{(2)}|n_2 > 0) &= E\left(\hat{\theta}_{(2)}|\hat{\theta}_1 < \sqrt{\frac{c^2}{w_1}} - \delta\right) \\ &= \theta + \frac{w_1}{c^2}E\left\{(\hat{\theta}_1 - \theta)(\hat{\theta}_1 + \delta)^2\middle|\hat{\theta}_1 < \sqrt{\frac{c^2}{w_1}} - \delta\right\} \\ &\le \theta + \frac{w_1}{c^2}\frac{E\{(\hat{\theta}_1 - \theta)(\hat{\theta}_1 + \delta)^2\}}{\Phi(h)} \\ &= \theta + \frac{2(\theta + \delta)}{c^2\Phi(h)}. \end{aligned} \tag{20}$$

# B Additional Materials for Section 2

| $\theta$ | $E[\hat{\theta}_{(2)}]$ | | | | $E_2$ | | | | $d_2$ | | | |
|---|---|---|---|---|---|---|---|---|---|---|---|---|
| | $\tau^2 = 0.00$ | $\tau^2 = 0.02$ | $\tau^2 = 0.04$ | $\tau^2 = 0.06$ | $\tau^2 = 0.00$ | $\tau^2 = 0.02$ | $\tau^2 = 0.04$ | $\tau^2 = 0.06$ | $d_{21}$ | $d_{22}$ | $d_{23}$ | $d_{24}$ |
| 0.00 | -0.0120 | -0.0071 | - 0.0146 | -0.0142 | 0.1865 | 0.1968 | 0.1990 | 0.2145 | 624 | 651 | 628 | 660 |
| 0.05 | 0.0395 | 0.0396 | 0.0446 | 0.0447 | 0.2239 | 0.2321 | 0.2260 | 0.1991 | 681 | 689 | 717 | 690 |
| 0.10 | 0.0944 | 0.0887 | 0.0863 | 0.0923 | 0.2689 | 0.2543 | 0.2421 | 0.2509 | 744 | 738 | 669 | 725 |
| 0.15 | 0.1458 | 0.1427 | 0.1393 | 0.1326 | 0.2564 | 0.2642 | 0.2585 | 0.2567 | 746 | 783 | 793 | 721 |
| 0.20 | 0.1933 | 0.11895 | 0.2008 | 0.1986 | 0.21893 | 0.2850 | 0.3100 | 0.2823 | 866 | 783 | 766 | 728 |
| 0.25 | 0.2304 | 0.2343 | 0.2606 | 0.2467 | 0.3113 | 0.3128 | 0.3446 | 0.3217 | 815 | 801 | 792 | 749 |
| 0.30 | 0.2883 | 0.2938 | 0.2864 | 0.2944 | 0.3496 | 0.3537 | 0.3608 | 0.3492 | 860 | 834 | 804 | 777 |
| 0.35 | 0.3464 | 0.3460 | 0.3519 | 0.3511 | 0.3891 | 0.3681 | 0.3552 | 0.3717 | 837 | 864 | 852 | 815 |
| 0.40 | 0.4115 | 0.3932 | 0.3989 | 0.3996 | 0.3818 | 0.4106 | 0.3781 | 0.3923 | 822 | 825 | 829 | 815 |
| 0.45 | 0.4611 | 0.4506 | 0.4438 | 0.4674 | 0.4257 | 0.4390 | 0.4307 | 0.4288 | 875 | 886 | 816 | 778 |
| 0.50 | 0.5030 | 0.4978 | 0.5002 | 0.5038 | 0.4377 | 0.4335 | 0.4501 | 0.4475 | 850 | 803 | 851 | 781 |
| 0.55 | 0.5564 | 0.5595 | 0.5508 | 0.5485 | 0.5048 | 0.4858 | 0.4760 | 0.4582 | 817 | 804 | 785 | 768 |
| 0.60 | 0.6071 | 0.6110 | 0.6037 | 0.6014 | 0.5085 | 0.4925 | 0.5030 | 0.4976 | 825 | 744 | 751 | 749 |
| 0.65 | 0.6642 | 0.6487 | 0.6538 | 0.6539 | 0.5506 | 0.5325 | 0.5388 | 0.5317 | 799 | 793 | 725 | 693 |
| 0.70 | 0.7025 | 0.7080 | 0.7097 | 0.7036 | 0.5529 | 0.5622 | 0.5737 | 0.5565 | 755 | 751 | 724 | 717 |
| 0.75 | 0.7634 | 0.7542 | 0.7625 | 0.7607 | 0.5841 | 0.5722 | 0.5805 | 0.5803 | 722 | 684 | 677 | 677 |
| 0.80 | 0.8098 | 0.8116 | 0.8075 | 0.8212 | 0.6021 | 0.6257 | 0.5852 | 0.6057 | 688 | 689 | 635 | 643 |
| 0.85 | 0.8565 | 0.8609 | 0.87705 | 0.8658 | 0.5981 | 0.6285 | 0.6279 | 0.6444 | 671 | 625 | 615 | 624 |
| 0.90 | 0.9197 | 0.9207 | 0.9100 | 0.9087 | 0.6598 | 0.6560 | 0.6532 | 0.6319 | 598 | 577 | 584 | 593 |
| 0.95 | 0.9592 | 0.9570 | 0.9706 | 0.9605 | 0.6825 | 0.6724 | 0.6861 | 0.6640 | 519 | 560 | 560 | 528 |

Table 1: Unconditional and conditional expected values of the estimated cumulative effects $\hat{\theta}_{(2)}$ obtained from $N = 10000$ simulated values of $\hat{\theta}_1$ from $N(\theta, \sigma^2 + \tau^2)$ distribution with $\theta = 0(0.05)0.95$ and $\tau^2 = (0.00, 0.02, 0.04, 0.06)$ using equal weights $\omega_1 = \omega_2$ and the variance $\sigma^2 = 19.94$, sample size $n = 60$ (corresponding to the within-study variance $s_1^2 = 19.94$ and sample size of $n_1 = 60$ in Example of Section 4), under the power-law model with $\theta_0 = 0.5$ and $t = 3$. Here $E[\hat{\theta}_{(2)}]$ is the unconditional mean, $E_2$ is the conditional mean and $d_2$ is the number of new studies at the 2nd step (given 10,000 studies at step 1)

| $\theta$ | $E[\hat{\theta}_3]$ | | | | $E_3$ | | | | $d_3$ | | | |
|---|---|---|---|---|---|---|---|---|---|---|---|---|
| | $\tau^2 = 0.00$ | $\tau^2 = 0.02$ | $\tau^2 = 0.04$ | $\tau^2 = 0.06$ | $\tau^2 = 0.00$ | $\tau^2 = 0.02$ | $\tau^2 = 0.04$ | $\tau^2 = 0.06$ | $d_{31}$ | $d_{32}$ | $d_{33}$ | $d_{34}$ |
| 0.00 | -0.0132 | -0.0083 | -0.0160 | -0.0152 | 0.2544 | 0.2393 | 0.2315 | 0.2749 | 88 | 89 | 85 | 93 |
| 0.05 | 0.0383 | 0.0382 | 0.0432 | 0.0435 | 0.2722 | 0.2560 | 0.2564 | 0.2358 | 97 | 92 | 106 | 81 |
| 0.10 | 0.0934 | 0.0877 | 0.0855 | 0.0914 | 0.3087 | 0.2791 | 0.2940 | 0.2972 | 123 | 86 | 88 | 96 |
| 0.15 | 0.1446 | 0.1416 | 0.1386 | 0.1323 | 0.2944 | 0.2913 | 0.3249 | 0.3621 | 117 | 114 | 112 | 105 |
| 0.20 | 0.1920 | 0.1884 | 0.1999 | 0.1981 | 0.3094 | 0.3137 | 0.3242 | 0.3236 | 137 | 126 | 117 | 100 |
| 0.25 | 0.2300 | 0.2338 | 0.2598 | 0.2462 | 0.3710 | 0.3551 | 0.3361 | 0.3509 | 115 | 134 | 115 | 120 |
| 0.30 | 0.2870 | 0.2935 | 0.2860 | 0.2942 | 0.3195 | 0.3626 | 0.3663 | 0.3797 | 155 | 114 | 126 | 106 |
| 0.35 | 0.3465 | 0.3455 | 0.3514 | 0.3506 | 0.4100 | 0.3727 | 0.3683 | 0.3627 | 123 | 131 | 128 | 123 |
| 0.40 | 0.4117 | 0.3937 | 0.3988 | 0.3995 | 0.4149 | 0.4358 | 0.4047 | 0.3927 | 135 | 128 | 123 | 150 |
| 0.45 | 0.4611 | 0.4510 | 0.4437 | 0.4677 | 0.4149 | 0.4358 | 0.4047 | 0.4213 | 147 | 130 | 126 | 117 |
| 0.50 | 0.5031 | 0.4983 | 0.5006 | 0.5044 | 0.3999 | 0.4358 | 0.4441 | 0.4647 | 155 | 140 | 125 | 115 |
| 0.55 | 0.5567 | 0.5600 | 0.5513 | 0.5494 | 0.4318 | 0.4385 | 0.4582 | 0.4740 | 134 | 129 | 129 | 116 |
| 0.60 | 0.6085 | 0.6117 | 0.6044 | 0.6021 | 0.5100 | 0.4707 | 0.4677 | 0.4758 | 147 | 124 | 109 | 101 |
| 0.65 | 0.6650 | 0.6498 | 0.6546 | 0.6545 | 0.4770 | 0.5044 | 0.4880 | 0.4585 | 136 | 116 | 96 | 100 |
| 0.70 | 0.7039 | 0.7089 | 0.7106 | 0.7046 | 0.5501 | 0.4982 | 0.5032 | 0.5135 | 108 | 102 | 105 | 99 |
| 0.75 | 0.7646 | 0.7552 | 0.7637 | 0.7613 | 0.5563 | 0.5063 | 0.5432 | 0.4836 | 95 | 111 | 94 | 90 |
| 0.80 | 0.8112 | 0.8128 | 0.8086 | 0.8226 | 0.5805 | 0.5488 | 0.5383 | 0.5738 | 84 | 80 | 81 | 82 |
| 0.85 | 0.8574 | 0.8622 | 0.8718 | 0.8666 | 0.5311 | 0.5539 | 0.5769 | 0.5378 | 74 | 96 | 77 | 69 |
| 0.90 | 0.9207 | 0.9220 | 0.9106 | 0.9101 | 0.5541 | 0.5748 | 0.5144 | 0.5824 | 68 | 73 | 63 | 86 |
| 0.95 | 0.9604 | 0.9581 | 0.9713 | 0.9616 | 0.6463 | 0.6004 | 0.5631 | 0.6277 | 58 | 66 | 49 | 54 |

Table 2: Unconditional and conditional expected values of the estimated cumulative effects $\hat{\theta}_{(3)}$ obtained from $N = 10000$ simulated values of $\hat{\theta}_1$ from $N(\theta, \sigma^2 + \tau^2)$ distribution with $\theta = 0(0.05)0.95$ and $\tau^2 = (0.00, 0.02, 0.04, 0.06)$ using equal weights $\omega_1 = \omega_2 = \omega_3$ and the variance $\sigma^2 = 19.94$, sample size $n = 60$ (corresponding to the within-study variance $s_1^2 = 19.94$ and sample size of $n_1 = 60$ in Example of Section 4), under the power-law model with $\theta_0 = 0.5$ and $t = 3$. Here $E[\hat{\theta}_{(3)}]$ is the unconditional mean, $E_3$ is the conditional mean and $d_3$ is the number of new studies at the 3nd step (given 10,000 studies at step 1)

| $\theta$ | $E[\hat{\theta}_{(2)}]$ | | | | $E_2$ | | | | $d_2$ | | | |
|---|---|---|---|---|---|---|---|---|---|---|---|---|
| | $\tau^2 = 0.00$ | $\tau^2 = 0.02$ | $\tau^2 = 0.04$ | $\tau^2 = 0.06$ | $\tau^2 = 0.00$ | $\tau^2 = 0.02$ | $\tau^2 = 0.04$ | $\tau^2 = 0.06$ | $d_{21}$ | $d_{22}$ | $d_{23}$ | $d_{24}$ |
| 0.00 | 0.0554 | 0.0578 | 0.0599 | 0.0563 | -0.0788 | -0.0718 | -0.0781 | -0.0823 | 8076 | 8166 | 8033 | 8076 |
| 0.05 | 0.1079 | 0.1077 | 0.1221 | 0.1211 | -0.0202 | -0.0262 | -0.0247 | -0.0270 | 7856 | 7889 | 7978 | 7821 |
| 0.10 | 0.1616 | 0.1560 | 0.1689 | 0.1722 | 0.0223 | 0.0183 | 0.0149 | 0.0067 | 7787 | 7878 | 7526 | 7554 |
| 0.15 | 0.2144 | 0.2150 | 0.2209 | 0.2188 | 0.0680 | 0.0643 | 0.0628 | 0.0597 | 7595 | 7604 | 7362 | 7330 |
| 0.20 | 0.2655 | 0.2679 | 0.2728 | 0.2716 | 0.1178 | 0.1088 | 0.1085 | 0.0947 | 7479 | 7398 | 7076 | 7138 |
| 0.25 | 0.3100 | 0.3117 | 0.3356 | 0.3244 | 0.1528 | 0.1481 | 0.1548 | 0.1483 | 7290 | 7309 | 7075 | 7024 |
| 0.30 | 0.3658 | 0.3683 | 0.3670 | 0.3722 | 0.1934 | 0.1932 | 0.1931 | 0.1913 | 7015 | 7025 | 6729 | 6845 |
| 0.35 | 0.4169 | 0.4171 | 0.4274 | 0.4310 | 0.2412 | 0.2313 | 0.2384 | 0.2472 | 6810 | 6876 | 6676 | 6642 |
| 0.40 | 0.4795 | 0.4751 | 0.4779 | 0.4768 | 0.2862 | 0.2926 | 0.2909 | 0.2797 | 6501 | 6597 | 6397 | 6367 |
| 0.45 | 0.5284 | 0.5221 | 0.5258 | 0.5440 | 0.3371 | 0.3268 | 0.3310 | 0.3302 | 6417 | 6465 | 6162 | 6184 |
| 0.50 | 0.5704 | 0.5766 | 0.5717 | 0.5782 | 0.3778 | 0.3805 | 0.3680 | 0.3660 | 6276 | 6270 | 6059 | 6051 |
| 0.55 | 0.6289 | 0.6314 | 0.6260 | 0.6261 | 0.4215 | 0.4244 | 0.4166 | 0.4130 | 5915 | 5898 | 5731 | 5851 |
| 0.60 | 0.6748 | 0.6816 | 0.6737 | 0.6810 | 0.4667 | 0.4616 | 0.4976 | 0.4685 | 5728 | 5743 | 5558 | 5578 |
| 0.65 | 0.7265 | 0.7215 | 0.7249 | 0.7269 | 0.5105 | 0.5178 | 0.5024 | 0.4963 | 5522 | 5653 | 5352 | 5354 |
| 0.70 | 0.7677 | 0.7761 | 0.7765 | 0.7768 | 0.5523 | 0.5557 | 0.5514 | 0.5440 | 5282 | 5366 | 5128 | 5204 |
| 0.75 | 0.8290 | 0.8211 | 0.8265 | 0.8288 | 0.6109 | 0.5974 | 0.5909 | 0.5880 | 5144 | 5118 | 4935 | 4889 |
| 0.80 | 0.8290 | 0.8735 | 0.8795 | 0.8895 | 0.6401 | 0.6441 | 0.6388 | 0.6367 | 4758 | 4884 | 4684 | 4712 |
| 0.85 | 0.9205 | 0.9202 | 0.9311 | 0.9304 | 0.6886 | 0.6795 | 0.6818 | 0.6818 | 4628 | 4603 | 4454 | 4533 |
| 0.90 | 0.9754 | 0.9756 | 0.9751 | 0.9751 | 0.7407 | 0.7293 | 0.7274 | 0.7272 | 4387 | 4375 | 4216 | 4358 |
| 0.95 | 1.0152 | 1.0188 | 1.0284 | 1.0249 | 0.7694 | 0.7644 | 0.7765 | 0.7632 | 4119 | 4231 | 4122 | 4108 |

Table 3: Unconditional and conditional expected values of the estimated cumulative effects $\hat{\theta}_{(2)}$ obtained from $N = 10000$ simulated values of $\hat{\theta}_1$ from $N(\theta, \sigma^2 + \tau^2)$ distribution with $\theta = 0(0.05)0.95$ and $\tau^2 = (0.00, 0.02, 0.04, 0.06)$ using equal weights $\omega_1 = \omega_2$ and the variance $\sigma^2 = 19.94$, sample size $n = 60$ (corresponding to the within-study variance $s_1^2 = 19.94$ and sample size of $n_1 = 60$ in Example of Section 4), under the extreme value model with $\theta_0 = 0.5$. Here $E[\hat{\theta}_{(2)}]$ is the unconditional mean, $E_2$ is the conditional mean and $d_2$ is the number of new studies at the 2nd step (given 10,000 studies at step 1)

| $\theta$ | $E[\hat{\theta}_3]$ | | | | $E_3$ | | | | $d_3$ | | | |
|---|---|---|---|---|---|---|---|---|---|---|---|---|
| | $\tau^2 = 0.00$ | $\tau^2 = 0.02$ | $\tau^2 = 0.04$ | $\tau^2 = 0.06$ | $\tau^2 = 0.00$ | $\tau^2 = 0.02$ | $\tau^2 = 0.04$ | $\tau^2 = 0.06$ | $d_{31}$ | $d_{32}$ | $d_{33}$ | $d_{34}$ |
| 0.00 | 0.0864 | 0.886 | 0.0908 | 0.0916 | -0.0880 | -0.0840 | -0.0924 | -0.0903 | 7155 | 7189 | 7062 | 7077 |
| 0.05 | 0.1384 | 0.1420 | 0.1530 | 0.1578 | -0.0364 | -0.0395 | -0.0436 | -0.0421 | 6966 | 6925 | 6751 | 6708 |
| 0.10 | 0.1935 | 0.1899 | 0.2010 | 0.2067 | 0.0058 | 0.0020 | -0.0038 | -0.0150 | 6629 | 6647 | 6511 | 6431 |
| 0.15 | 0.2474 | 0.2483 | 0.2542 | 0.2581 | 0.0479 | 0.454 | 0.0362 | 0.0453 | 6304 | 6287 | 6169 | 16214 |
| 0.20 | 0.2977 | 0.3000 | 0.3089 | 0.3071 | 0.0982 | 0.0858 | 0.0852 | 0.0717 | 6073 | 5978 | 5939 | 5930 |
| 0.25 | 0.3429 | 0.3444 | 0.3691 | 0.3586 | 0.1278 | 0.1262 | 0.1239 | 0.1230 | 5807 | 5811 | 5569 | 5660 |
| 0.30 | 0.4017 | 0.4025 | 0.4009 | 0.40912 | 0.1739 | 0.1707 | 0.1658 | 0.1659 | 5499 | 5464 | 5454 | 5441 |
| 0.35 | 0.4515 | 0.4494 | 0.4636 | 0.4668 | 0.2158 | 0.2005 | 0.2124 | 0.2135 | 5158 | 5210 | 5051 | 5069 |
| 0.40 | 0.5132 | 0.5095 | 0.5126 | 0.5139 | 0.2576 | 0.2661 | 0.2591 | 0.2498 | 4803 | 4803 | 4830 | 4859 |
| 0.45 | 0.5615 | 0.5557 | 0.5591 | 0.5784 | 0.3098 | 0.2934 | 0.2897 | 0.2933 | 4596 | 4649 | 4522 | 4487 |
| 0.50 | 0.6017 | 0.6094 | 0.6066 | 0.6132 | 0.3399 | 0.3395 | 0.3310 | 0.3326 | 4369 | 4298 | 4208 | 4260 |
| 0.55 | 0.6605 | 0.6624 | 0.6592 | 0.6624 | 0.3900 | 0.3861 | 0.3724 | 0.3780 | 3955 | 3909 | 4001 | 4075 |
| 0.60 | 0.7045 | 0.7111 | 0.7083 | 0.7127 | 0.4320 | 0.4225 | 0.4149 | 0.4201 | 3730 | 3715 | 3722 | 3760 |
| 0.65 | 0.7560 | 0.7515 | 0.7593 | 0.7614 | 0.4762 | 0.4689 | 0.4716 | 0.4585 | 3467 | 3454 | 3548 | 3540 |
| 0.70 | 0.7965 | 0.8017 | 0.8066 | 0.8093 | 0.5128 | 0.4961 | 0.5053 | 0.5043 | 3193 | 3198 | 3205 | 3264 |
| 0.75 | 0.8571 | 0.8511 | 0.8555 | 0.8581 | 0.5693 | 0.5477 | 0.5438 | 0.5438 | 2918 | 2988 | 2952 | 3022 |
| 0.80 | 0.8958 | 0.9007 | 0.9086 | 0.9177 | 0.6077 | 0.5963 | 0.5930 | 0.5861 | 2633 | 2691 | 2788 | 2743 |
| 0.85 | 0.9447 | 0.9471 | 0.9591 | 0.9584 | 0.6397 | 0.6368 | 0.6293 | 0.6251 | 2422 | 2464 | 2478 | 2496 |
| 0.90 | 0.9975 | 0.9986 | 1.0001 | 0.9994 | 0.6900 | 0.6759 | 0.6758 | 0.6818 | 2200 | 2240 | 2288 | 2388 |
| 0.95 | 1.0397 | 1.0425 | 1.0527 | 1.0524 | 0.7301 | 0.7122 | 0.7210 | 0.7153 | 1979 | 2061 | 2116 | 2221 |

Table 4: Unconditional and conditional expected values of the estimated cumulative effects $\hat{\theta}_{(3)}$ obtained from $N = 10000$ simulated values of $\hat{\theta}_1$ from $N(\theta, \sigma^2 + \tau^2)$ distribution with $\theta = 0(0.05)0.95$ and $\tau^2 = (0.00, 0.02, 0.04, 0.06)$ using equal weights $\omega_1 = \omega_2 = \omega_3$ and the variance $\sigma^2 = 19.94$, sample size $n = 60$ (corresponding to the within-study variance $s_1^2 = 19.94$ and sample size of $n_1 = 60$ in Example of Section 4), under the extreme value model with $\theta_0 = 0.5$. Here $E[\hat{\theta}_{(3)}]$ is the unconditional mean, $E_3$ is the conditional mean and $d_3$ is the number of new studies at the 3th step (given 10,000 studies at step 1).

| $\theta$ | $E[\hat{\theta}_{(2)}]$ | | | | $E_2$ | | | | $d_2$ | | | |
|---|---|---|---|---|---|---|---|---|---|---|---|---|
| | $\tau^2 = 0.00$ | $\tau^2 = 0.02$ | $\tau^2 = 0.04$ | $\tau^2 = 0.06$ | $\tau^2 = 0.00$ | $\tau^2 = 0.02$ | $\tau^2 = 0.04$ | $\tau^2 = 0.06$ | $d_{21}$ | $d_{22}$ | $d_{23}$ | $d_{24}$ |
| 0.00 | 0.0558 | 0.0607 | 0.0597 | 0.0663 | -0.0743 | -0.0693 | -0.0758 | -0.0784 | 8105 | 8103 | 8064 | 7993 |
| 0.05 | 0.1114 | 0.1090 | 0.1192 | 0.1203 | -0.0173 | -0.0271 | -0.0207 | -0.0323 | 7985 | 7978 | 7951 | 7821 |
| 0.10 | 0.1603 | 0.1635 | 0.1608 | 0.1762 | 0.0225 | 0.0176 | 0.0060 | 0.0150 | 7821 | 7772 | 7758 | 7680 |
| 0.15 | 0.2187 | 0.2164 | 0.2200 | 0.2176 | 0.0671 | 0.0660 | 0.0634 | 0.0534 | 7585 | 7635 | 7585 | 7527 |
| 0.20 | 0.2652 | 0.2699 | 0.2768 | 0.2745 | 0.1138 | 0.1145 | 0.1091 | 0.1024 | 7494 | 7483 | 7341 | 7369 |
| 0.25 | 0.3138 | 0.3137 | 0.3331 | 0.3230 | 0.1565 | 0.1504 | 0.1492 | 0.1465 | 72305 | 7299 | 7101 | 7153 |
| 0.30 | 0.3686 | 0.3706 | 0.3610 | 0.3770 | 0.1982 | 0.1953 | 0.1855 | 0.1901 | 7047 | 6990 | 7058 | 6944 |
| 0.35 | 0.4196 | 0.4247 | 0.4314 | 0.4319 | 0.2508 | 0.2437 | 0.2431 | 0.2443 | 6845 | 6868 | 6788 | 6719 |
| 0.40 | 0.4806 | 0.4689 | 0.4749 | 0.4811 | 0.2885 | 0.2858 | 0.2791 | 0.2816 | 6518 | 6649 | 6559 | 6508 |
| 0.45 | 0.5275 | 0.5238 | 0.5252 | 0.5406 | 0.3319 | 0.3217 | 0.3315 | 0.3319 | 6348 | 6361 | 6433 | 6340 |
| 0.50 | 0.5779 | 0.5768 | 0.5776 | 0.5816 | 0.3846 | 0.3699 | 0.3740 | 0.3743 | 6246 | 6119 | 6112 | 6263 |
| 0.55 | 0.6238 | 0.6264 | 0.6356 | 0.6286 | 0.4148 | 0.4165 | 0.4243 | 0.4102 | 5928 | 5942 | 5966 | 5977 |
| 0.60 | 0.6759 | 0.6807 | 0.6719 | 0.6785 | 0.4660 | 0.4613 | 0.4476 | 0.4653 | 5730 | 5556 | 5642 | 5599 |
| 0.65 | 0.7310 | 0.7209 | 0.7239 | 0.7319 | 0.5234 | 0.5099 | 0.4999 | 0.5070 | 5530 | 5556 | 5642 | 5599 |
| 0.70 | 0.7682 | 0.7767 | 0.7770 | 0.7781 | 0.5578 | 0.5605 | 0.5466 | 0.5444 | 5338 | 5359 | 5383 | 5389 |
| 0.75 | 0.8192 | 0.8205 | 0.8320 | 0.8305 | 0.6024 | 0.5847 | 0.5914 | 0.5836 | 5065 | 5051 | 5125 | 5019 |
| 0.80 | 0.8706 | 0.8722 | 0.8758 | 0.8881 | 0.6468 | 0.6444 | 0.6294 | 0.6395 | 4821 | 4831 | 4918 | 4857 |
| 0.85 | 0.9189 | 0.9230 | 0.9361 | 0.9314 | 0.6916 | 0.6823 | 0.6913 | 0.6787 | 4673 | 4690 | 4682 | 4696 |
| 0.90 | 0.9792 | 0.9779 | 0.9761 | 0.9314 | 0.7426 | 0.7303 | 0.7245 | 0.7340 | 4340 | 4297 | 4276 | 4526 |
| 0.95 | 1.0154 | 1.0166 | 1.0264 | 1.0254 | 0.7734 | 0.7697 | 0.7683 | 0.7602 | 4263 | 4243 | 4260 | 4299 |

Table 5: Unconditional and conditional expected values of the estimated cumulative effects $\hat{\theta}_{(2)}$ obtained from $N = 10000$ simulated values of $\hat{\theta}_1$ from $N(\theta, \sigma^2 + \tau^2)$ distribution with $\theta = 0(0.05)0.95$ and $\tau^2 = (0.00, 0.02, 0.04, 0.06)$ using equal weights $\omega_1 = \omega_2$ and the variance $\sigma^2 = 19.94$, sample size $n = 60$ (corresponding to the within-study variance $s_1^2 = 19.94$ and sample size of $n_1 = 60$ in Example of Section 4), under the probit model with $\theta_0 = 0.5$, $\alpha = 0$ and $\beta = 1$. Here $E[\hat{\theta}_{(2)}]$ is the unconditional mean, $E_2$ is the conditional mean and $d_2$ is the number of new studies at the 2nd step (given 10,000 studies at step 1).

| $\theta$ | $\mathrm{E}[\hat{\theta}_3]$ | | | | $\mathrm{E}_3$ | | | | $d_3$ | | | |
|---|---|---|---|---|---|---|---|---|---|---|---|---|
| | $\tau^2 = 0.00$ | $\tau^2 = 0.02$ | $\tau^2 = 0.04$ | $\tau^2 = 0.06$ | $\tau^2 = 0.00$ | $\tau^2 = 0.02$ | $\tau^2 = 0.04$ | $\tau^2 = 0.06$ | $d_{31}$ | $d_{32}$ | $d_{33}$ | $d_{34}$ |
| 0.00 | 0.0475 | 0.0531 | 0.0511 | 0.0582 | 0.0678 | 0.0633 | 0.0710 | 0.0758 | 2367 | 2402 | 2390 | 2366 |
| 0.05 | 0.1060 | 0.1002 | 0.1093 | 0.1114 | 0.1239 | 0.1067 | 0.1211 | 0.1213 | 2487 | 2432 | 2468 | 2347 |
| 0.10 | 0.1558 | 0.1553 | 0.1553 | 0.1686 | 0.1408 | 0.1523 | 0.1532 | 1680 | 2590 | 2505 | 2503 | 2511 |
| 0.15 | 0.2131 | 0.2101 | 0.2114 | 0.2111 | 0.1969 | 0.1923 | 0.2092 | 0.2200 | 2594 | 2599 | 2532 | 2478 |
| 0.20 | 0.2611 | 0.2647 | 0.2706 | 0.2672 | 0.2376 | 0.2487 | 0.2430 | 0.2545 | 2658 | 2705 | 2642 | 2573 |
| 0.25 | 0.3102 | 0.3084 | 0.3267 | 0.3183 | 0.2839 | 0.2847 | 0.2847 | 0.2948 | 2658 | 2705 | 2642 | 2573 |
| 0.30 | 0.3659 | 0.3648 | 0.3560 | 0.3725 | 0.3231 | 0.3207 | 0.3172 | 0.3318 | 2686 | 2734 | 2582 | 2723 |
| 0.35 | 0.4184 | 0.4219 | 0.4278 | 0.4259 | 0.3819 | 0.3686 | 0.3828 | 0.3886 | 2705 | 2706 | 2680 | 2653 |
| 0.40 | 0.4779 | 0.4663 | 0.4730 | 0.4790 | 0.4059 | 0.4091 | 0.4192 | 0.4252 | 2859 | 2740 | 2715 | 2718 |
| 0.45 | 0.5273 | 0.5227 | 0.5247 | 0.5379 | 0.4513 | 0.4537 | 0.4656 | 0.4667 | 2761 | 2817 | 2684 | 2746 |
| 0.50 | 0.5778 | 0.5782 | 0.5774 | 0.5804 | 0.5102 | 0.5066 | 0.5007 | 0.5116 | 2753 | 2739 | 2758 | 2754 |
| 0.55 | 0.6258 | 0.6278 | 0.6353 | 0.6270 | 0.5427 | 0.5432 | 0.5476 | 0.5447 | 2858 | 2802 | 2758 | 2827 |
| 0.60 | 0.6778 | 0.6841 | 0.6744 | 0.6801 | 0.5845 | 0.5955 | 0.5781 | 0.5982 | 2691 | 2788 | 2796 | 2752 |
| 0.65 | 0.7352 | 0.7233 | 0.7258 | 0.7314 | 0.5845 | 0.5955 | 0.5781 | 0.5982 | 2818 | 2777 | 2709 | 2846 |
| 0.70 | 0.7726 | 0.7795 | 0.7794 | 0.7802 | 0.6419 | 0.6285 | 0.6397 | 0.6423 | 2880 | 2785 | 2777 | 2823 |
| 0.75 | 0.8229 | 0.8243 | 0.8365 | 0.8344 | 0.6835 | 0.6804 | 0.6809 | 0.6792 | 2761 | 2768 | 2845 | 2800 |
| 0.80 | 0.8748 | 0.8770 | 0.8811 | 0.8928 | 0.7204 | 0.7100 | 0.7194 | 0.7232 | 2752 | 2700 | 2732 | 2710 |
| 0.85 | 0.9244 | 0.9293 | 0.9402 | 0.9372 | 0.7720 | 0.7667 | 0.7598 | 0.7728 | 2645 | 2658 | 2719 | 2710 |
| 0.90 | 0.9849 | 0.9850 | 0.9807 | 0.9825 | 0.8080 | 0.8102 | 0.8139 | 0.8152 | 2714 | 2630 | 2730 | 2663 |
| 0.95 | 1.0216 | 1.0229 | 1.0351 | 1.0326 | 0.8883 | 0.8926 | 0.9075 | 0.8951 | 2560 | 2518 | 2532 | 2609 |

Table 6: Unconditional and conditional expected values of the estimated cumulative effects $\hat{\theta}_{(3)}$ obtained from $N = 10000$ simulated values of $\hat{\theta}_1$ from $N(\theta, \sigma^2 + \tau^2)$ distribution with $\theta = 0(0.05)0.95$ and $\tau^2 = (0.00, 0.02, 0.04, 0.06)$ using equal weights $\omega_1 = \omega_2 = \omega_3$ and the variance $\sigma^2 = 19.94$, sample size $n = 60$ (corresponding to the within-study variance $s_1^2 = 19.94$ and sample size of $n_1 = 60$ in Example of Section 4), under the probit model with $\theta_0 = 0.5$, $\alpha = 0$ and $\beta = 1$. Here $\mathrm{E}[\hat{\theta}_{(3)}]$ is the unconditional mean, $\mathrm{E}_3$ is the conditional mean and $d_3$ is the number of new studies at the 3th step (given 10,000 studies at step 1).

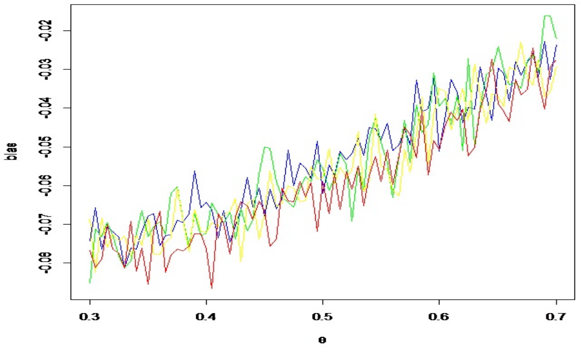


(a)

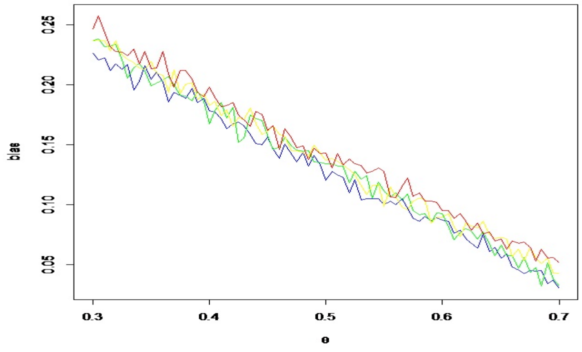


(b)

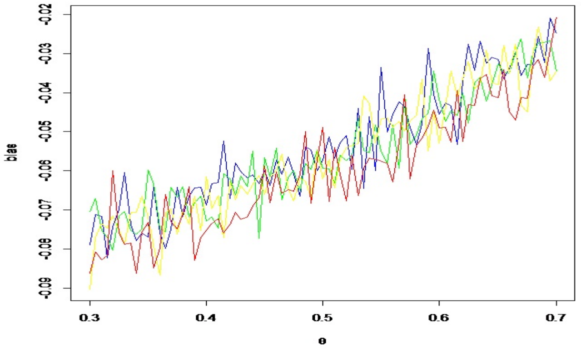


(c)

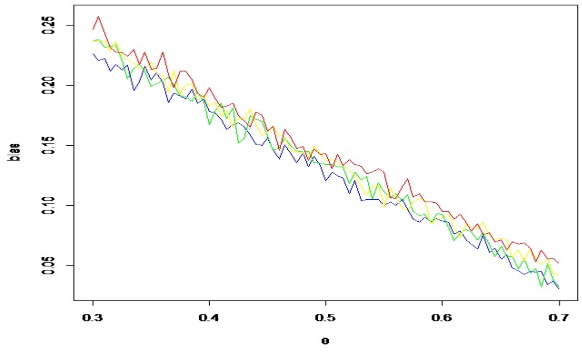


(d)

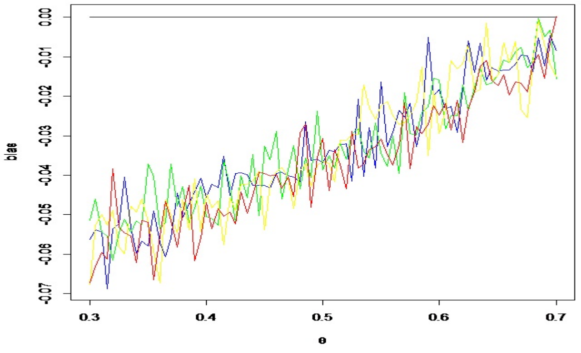


(e)

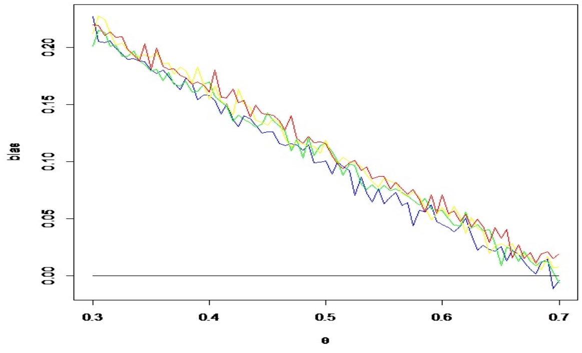


(f)

Figure S1: Biases of unconditional (left) and conditional (right) expected values of the cumulative effects $\hat{\theta}_{(3)}$ at the third study for the $\tau^2$ values of 0 (blue), 0.02 (green), 0.04 (yellow) and 0.06 (red). The rows 1 to 3 correspond to the biases under the power-law with $t = 3$, the extreme value and the probit with $\alpha = 0$ and $\beta = 1$ models, respectively. Results from 10000 simulations at each value of $\theta = 03(0.05)0.7$ for the target value of $\theta_0 = 0.5$, equal weights $\omega_1 = \omega_2 = \omega_3$ and the variance $\sigma^2 = 1/3$ (corresponding to the within-study variance $s_1^2 = 19.94$ and sample size of $n_1 = 61$ in Example of Section 4).

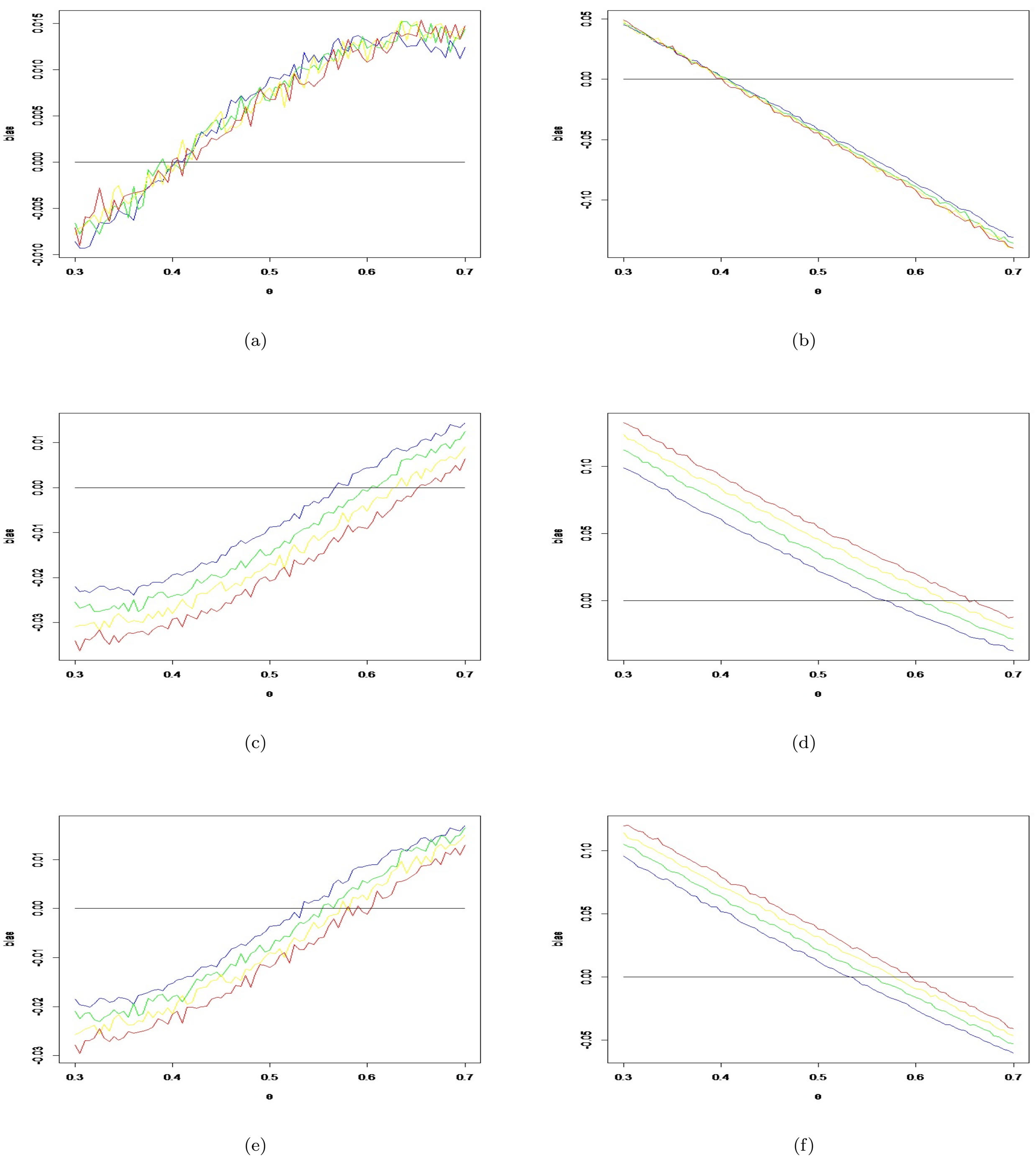


Figure S2: Biases of unconditional (left) and conditional (right) expected values of the cumulative effects $\hat{\theta}_{(2)}$ at the second study for the $\tau^2$ values of 0 (blue), 0.02 (green), 0.04 (yellow) and 0.06 (red). The rows 1 to 3 correspond to the biases under the power-law with $t = 3$, the extreme value and the probit with $\alpha = 0$ and $\beta = 1$ models, respectively. Results from 10000 simulations at each value of $\theta = 03(0.05)0.7$ for the target value of $\theta_0 = 0.5$, equal weights $\omega_1 = \omega_2$ and the variance $\sigma^2 = 0.04$ (corresponding to the within-study variance $s_1^2 = 19.94$ and sample size of $n_1 = 500$ in Example of Section 4). For the calculation of the probabilities using the probit; $\alpha = 0$ and $\beta = 1$.

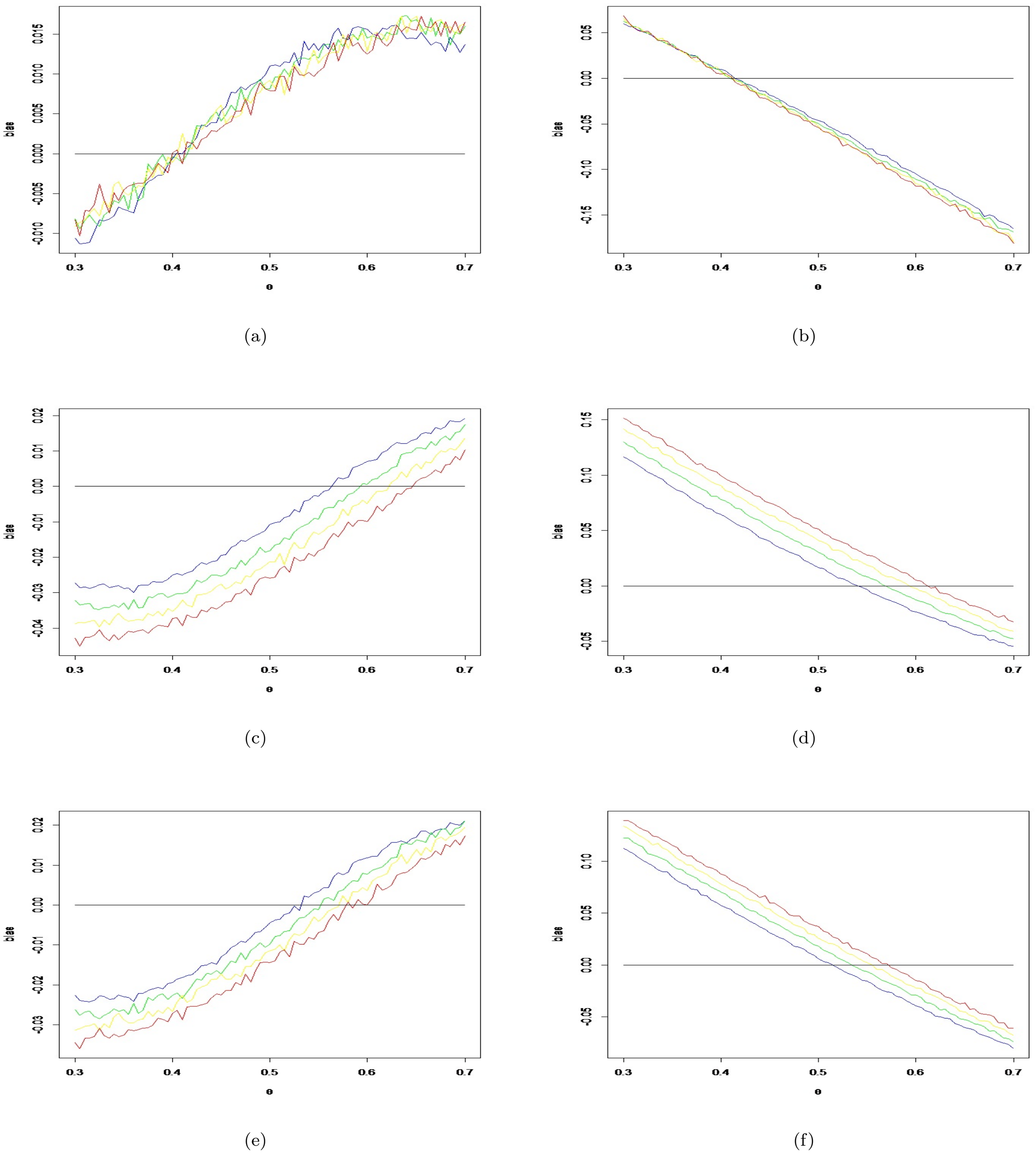


Figure S3: Biases of unconditional (left) and conditional (right) expected values of the cumulative effects $\hat{\theta}_{(3)}$ at the third study for the $\tau^2$ values of 0 (blue), 0.02 (green), 0.04 (yellow) and 0.06 (red). The rows 1 to 3 correspond to the biases under the power-law with $t = 3$, the extreme value and the probit with $\alpha = 0$ and $\beta = 1$ models, respectively. Results from 10000 simulations at each value of $\theta = 03(0.05)0.7$ for the target value of $\theta_0 = 0.5$, equal weights $\omega_1 = \omega_2 = \omega_3$ and the variance $\sigma^2 = 0.04$ (corresponding to the within-study variance $s_1^2 = 19.94$ and sample size of $n_1 = 500$ in Example of Section 4). For the calculation of the probabilities using the probit; $\alpha = 0$ and $\beta = 1$.

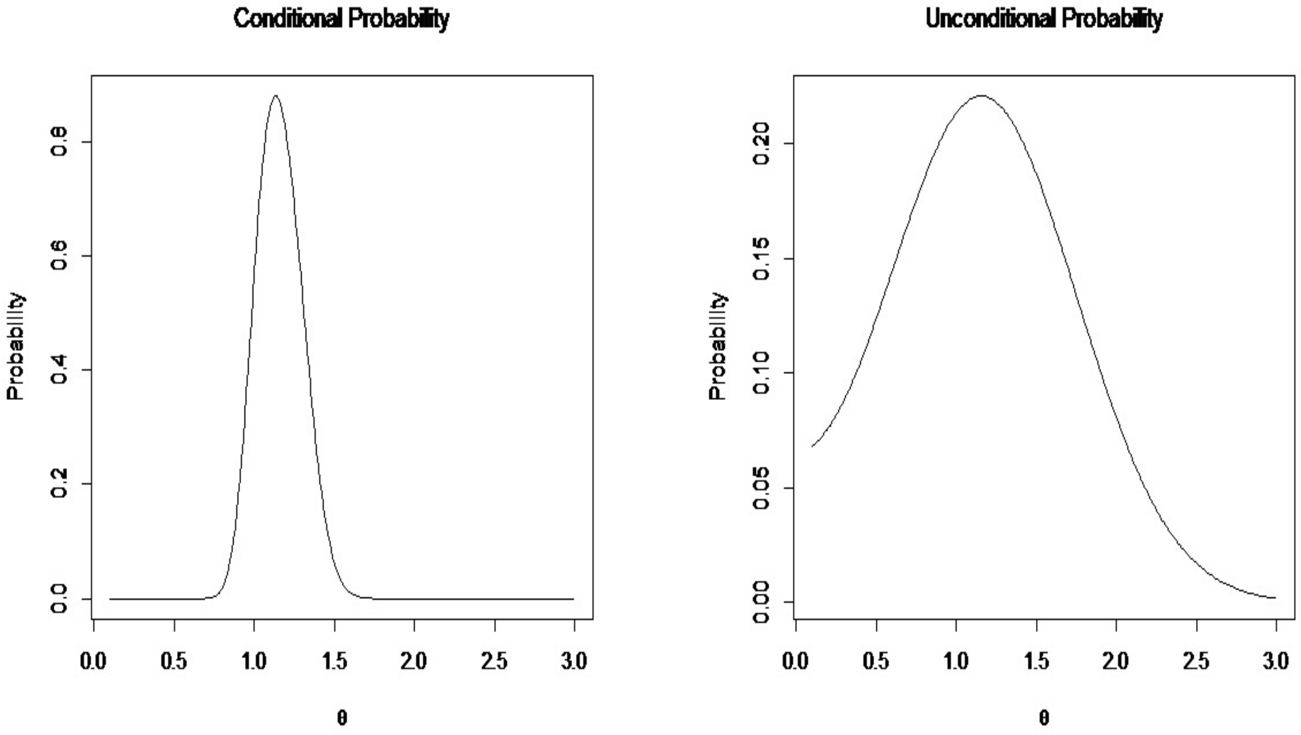


Figure S4: The conditional probability given that $\hat{\theta}_1 = \theta$ (left), and the unconditional probability (right) as a function of the true value $\theta$ of $\theta_0$ conducting the second trial for the power calculation rule of Section 2.2.2 with $n_1 = 60$, $\sigma_1^2 = 20$, $a = 30$ and $b = 80$.

# C Additional materials for Sections 3 and 4

The simulations for the sequential design bias for an arbitrary value of $d$ were designed as follows.

1. Simulate a trial with $n_1$ observations.

2. Let $\hat{\theta}_1$ be the sample mean and $\hat{\sigma}_1^2$ the sample variance. Compute $w_1 = n_1/\hat{\sigma}_1^2$.

3. For a given guess $\sigma_g^2$

   (a) compute
   $$n_2 = \left( \frac{c^2 \sigma_2^2}{(\hat{\theta}_1 + \delta)^2} - w_1 \right) \sigma_g^2.$$
   If $n_2 < 5$, replace it with $n_2 = 5$ and also set a maximum value of $n_2 = 1000$.

   (b) Simulate a second trial with $n_2$ observations. Let $\hat{\theta}_2$ be the sample mean and $\hat{\sigma}_2^2$ the sample variance.

(c) Compute $w_2 = n_2/\hat{\sigma}_2^2$ and

$$\hat{\theta}_{(2)} = \frac{w_1\hat{\theta}_1 + w_2\hat{\theta}_2}{w_1 + w_2}.$$

To evaluate a specific guess such as $\sigma_g^2 = \hat{\sigma}_1^2$ we carry out step 3 only once for each simulation. To evaluate the effect of different guesses, we repeat step 3 for the different values. As this required different sized trials in step 3 (b) for different guesses, this step may be repeated many times for each trial in step 1.

For our first simulations we took $\theta = 0.2$, $\delta = 0.2$, $\alpha = 0.05$ and $\beta = 0.2$. The variances $\sigma_1^2 = 19.94$, $\sigma_2^2 = 24.96$ and the sample size $n_1 = 61$ were taken from the example of a meta-analysis discussed in Section 4 so that $w_1 = 3.06$. Then $c^2/\theta^2 - w_1 = 136.22 > 0$. We conducted 1000 simulated initial experiments and took $d$ from 0.1 to 10 in steps of 0.1. Note that this required the simulation of 100 second trials in step 3 (b) for each initial trial, one for each value of $d$.

The plot the sequential design bias below is obtained by this algorithm for the data from the Example in Section 4.

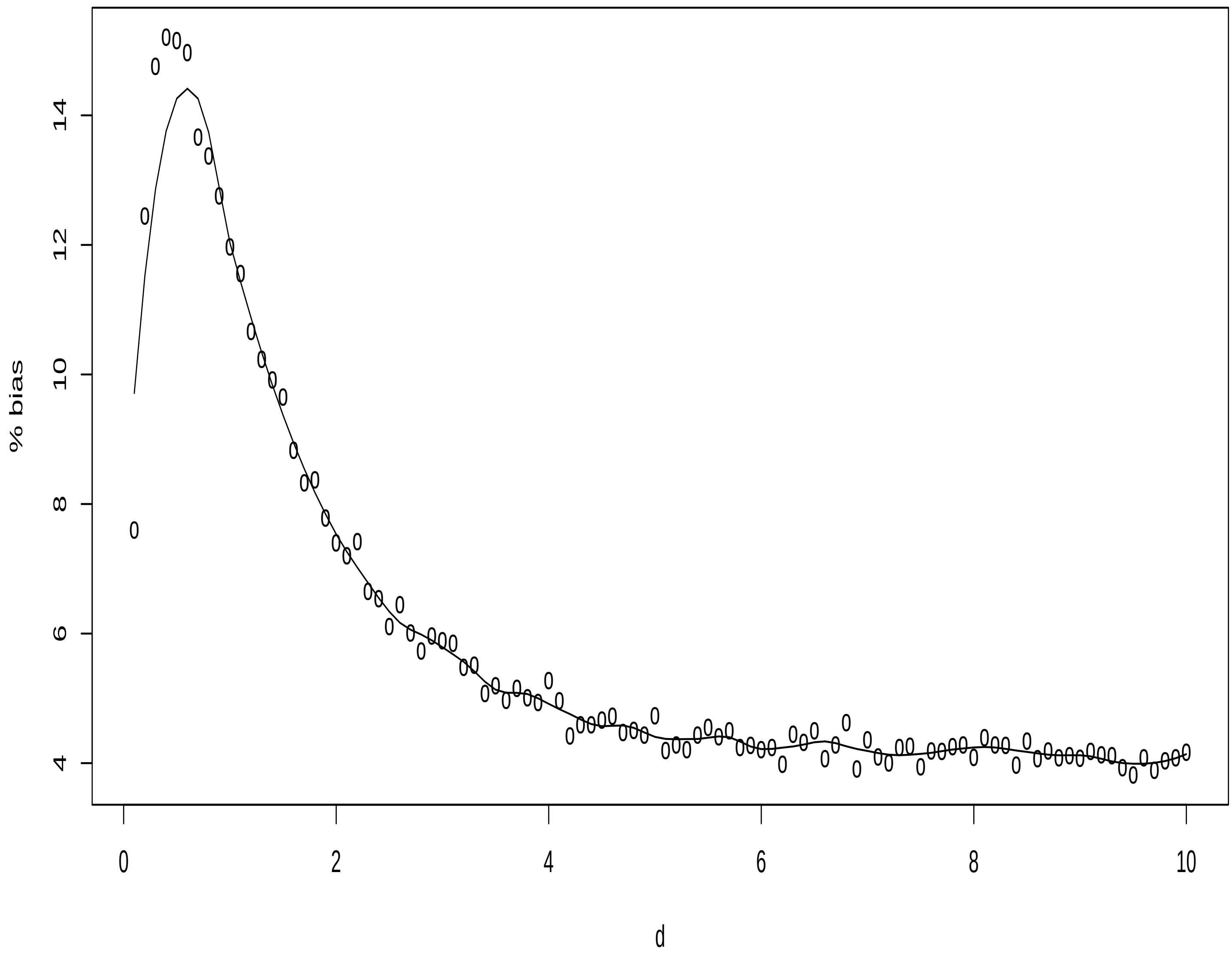


Figure S5: Plot of the percent bias against $d$ from 10000 simulations with $\theta = 0.86$, $\delta = -0.36$, $\alpha = 0.05$, $\beta = 0.2$, $\sigma_1^2 = 19.94$, $\sigma_2^2 = 24.96$ and $n_1 = 61$. The parameters were taken from the example of a meta-analysis discussed in Section 4. The locfit (Loader, 2012) package was used to smoothly estimate the mean.
